# Correction: Study of Protein-Protein Interactions in Septin Assembly: Multiple amphipathic helix domains cooperate in binding to the lipid membrane

**DOI:** 10.1371/journal.pcbi.1014812

**Published:** 2026-09-22

**Authors:** S. Mahsa Mofidi, Abhilash Sahoo, Christopher J. Edelmaier, Stephen J. Klawa, Ronit Freeman, Amy Gladfelter, M. Gregory Forest, Ehssan Nazockdast, Sonya M. Hanson

The following information is missing from the Funding statement: This work was supported by NSF MCB-2438870 to ASG and EN.
