# Supplementary material for: Study of Protein-Protein Interactions in Septin Assembly: Multiple amphipathic helix domains cooperate in binding to the lipid membrane
Source: PLoS Comput Biol. 2026 Apr 27;22(4):e1014230. doi: 10.1371/journal.pcbi.1014230 (PMC13143081; doi:10.1371/journal.pcbi.1014230)
Supplement: S1 File — (PDF) [file pcbi.1014230.s001.pdf]

**SUPPORTING MATERIAL**

**Table of Contents:**

The overall radius of curvature of the extended bound peptide for different replicas. The peptide keeps ~60 Å curvature over the simulations. ........................................................................3

Bending angle heat map for single-bound peptide in single and multiple systems for all replicas. 4

Average bending angle profile of the bound peptide over all four replicas in single- and two-peptide systems. ........................................................................5

(A) Z-component of the center of mass of a single unbound peptide's AH domain. The histogram is shown on the side for each replica (n=4). (B) Z-component of the center of mass of the unbound peptide's AH domain in the presence of a bound peptide (n = 8). The histogram is shown on the side. Replicas with no interaction are colored in gray. ........................................................................6

Violin and boxplot showing the median and deviation of the Z-component distance of the center of mass of the peptides from the membrane midline. (A) single unbound extended AH. (B) unbound extended AH in the presence of a bound peptide. ........................................................................7

Residue-resolved lipid contacts for the floating and bound peptides. Mean contact counts per simulation frame (± SEM across eight independent replicas) between each residue of the and membrane lipids, computed using a 4.5 Å distance cutoff. Total contacts (blue) report, for each residue, the mean number of *distinct PLPI (or DOPC) headgroup residues* whose headgroup atoms (P, O2–O6, and HO2–HO6) come within 4.5 Å of any atom of that residue. Charged contacts (orange) report the mean number of *distinct PLPI (or DOPC) headgroup residues* whose charged headgroup atoms (P and O2–O6) are within 4.5 Å of charged peptide side-chain atoms (Lys NZ; Arg NE/NH1/NH2; Asp OD1/OD2; Glu OE1/OE2; and His ND1/NE2 when present). (A) floating peptide–PLPI, (B) floating peptide–DOPC. ........................................................................8

Peptide-peptide contact maps reveal how a floating and a bound AH interact. (A)The zoomed-in inter-peptide contact map for other interacting replicas highlights how charged R residues of the N-terminal form salt bridges with the other peptide. (B) The domain-based coarse-grained contact maps of interacting replicas in different time windows show two peptides interacting in an anti-parallel configuration that reaches a steady state. ........................................................................9

Antiparallel-biased association of a bound and a floating peptide across eight replicas. (A) Time evolution of inter-peptide contact counts (heavy-atom distance < 4.5 Å). "Antiparallel" contacts (blue) are defined as contacts between the N-terminal patch (residues 1–8) of either peptide and the second half of the other peptide (residues 17–34). "Parallel" contacts (orange) are defined analogously using the C-terminal patch (residues 27–34) contacting the second half of the other peptide. (B) Replica persistence analysis based on cumulative time with contacts above threshold. For each replica, we computed the total time with antiparallel contacts ≥ 5 and parallel contacts ≥ 3, and plotted the number of replicas that maintain each criterion for at least X ns. (C)

Distribution of peptide–peptide orientation across all replicas for interacting frames (green) and non-interacting frames (red). The orientation metric is $\cos(\theta)$, where θ is the angle between the two peptide N to C axis vectors, each defined from the COM of the N-terminal patch (residues 1–8) to the COM of the C-terminal patch (residues 27–34) using heavy atoms. Interacting frames are enriched at negative $\cos(\theta)$, consistent with an antiparallel-like bias, whereas non-interacting frames sample a broader range of orientations. ........................................................10

number of contacts during simulation for every 8 replicas. In replicas 1, 3, 6, and 8, peptides could not find or interact with each other. For interacting peptides (replicas 2, 4, 5, 7), the figures show how charged residues are dominant in forming contacts. ..................................................12

Salt-bridge dynamics between the bound and floating peptides. (A)Time evolution of residue-residuer distances for the top-ranked charged pairs using the VMD salt-bridge analysis tool for representative interacting replicas (4, 5, and 7). (B) Mean salt-bridge occupancies across all eight replicas for the most frequently observed residue pairs, computed as the fraction of analyzed frames with a cutoff distance is ≤ 3.5 Å; error bars indicate the standard deviation across replicas. The highest-occupancy contacts involve R-E residue pairs (shown in yellow)...13

The tandem peptide remains stable when AH domains are arranged in an antiparallel configuration or extended with charged C-termini. (A) Simulation snapshots illustrate how tandem AH domains in an NC-NC arrangement hinge at the central linker, forming a stable antiparallel configuration. Sharp features in the contact map indicate strong, stable interactions between anti-parallel AH domains. (B) Tandem AH domains in a CN-NC (parallel) configuration exhibit fluctuating interactions, with the contact map showing an unstable state. (C) Extended Tandem AHs with added charged C-termini stabilize the parallel AHs in CN-NC configuration. (D-F) Helical content analysis reveals that stable states correspond to higher helical configurations. The NC configuration is shown in green, and the CN configuration is shown in purple for (D) anti-parallel, (E) parallel, and (F) extended parallel tandem AHs. .......................................................15

Final-frame snapshots of tandem AH simulations across independent replicates. (A) Tandem AH domains in NC-NC antiparallel arrangements, (B) Tandem AH domains in a CN-NC parallel arrangement, (C) Extended Tandem AH constructs with additional charged C-terminal domains to the parallel arrangement. ...........................................................................................16

Z-component of the center of mass distribution for peptides in simulations containing two membrane-bound peptides (n = 8), shown across different time windows. As the simulation progresses, both peptides exhibit a slight upward shift, indicating gradual displacement away from the membrane interior ..........................................................................................17

Side and top-view snapshots of simulations with three peptides at the beginning, 0.5, and 1 μs. (A) Replica 1, (B) Replica 2, (C) Replica 3. ........................................................................18

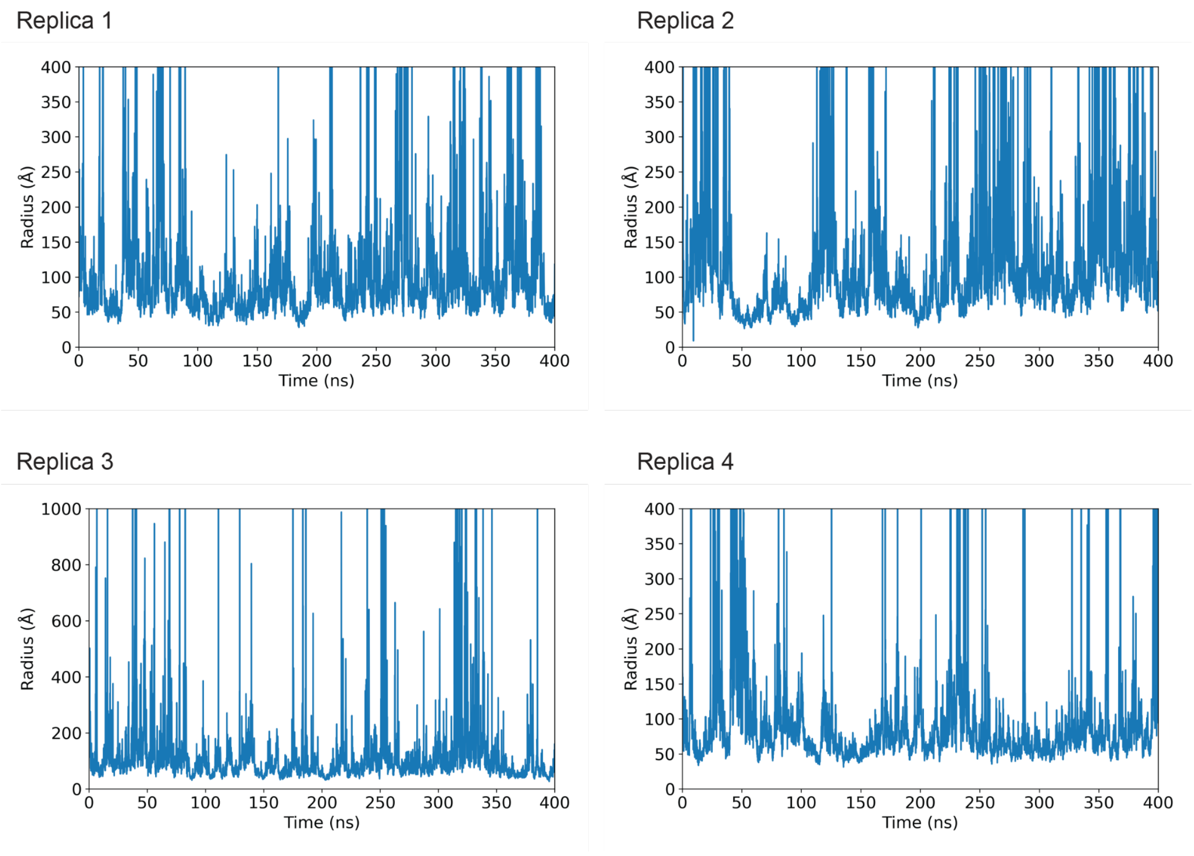


Fig A The overall radius of curvature of the extended bound peptide for different replicas. The peptide keeps ~60 Å curvature over the simulations.

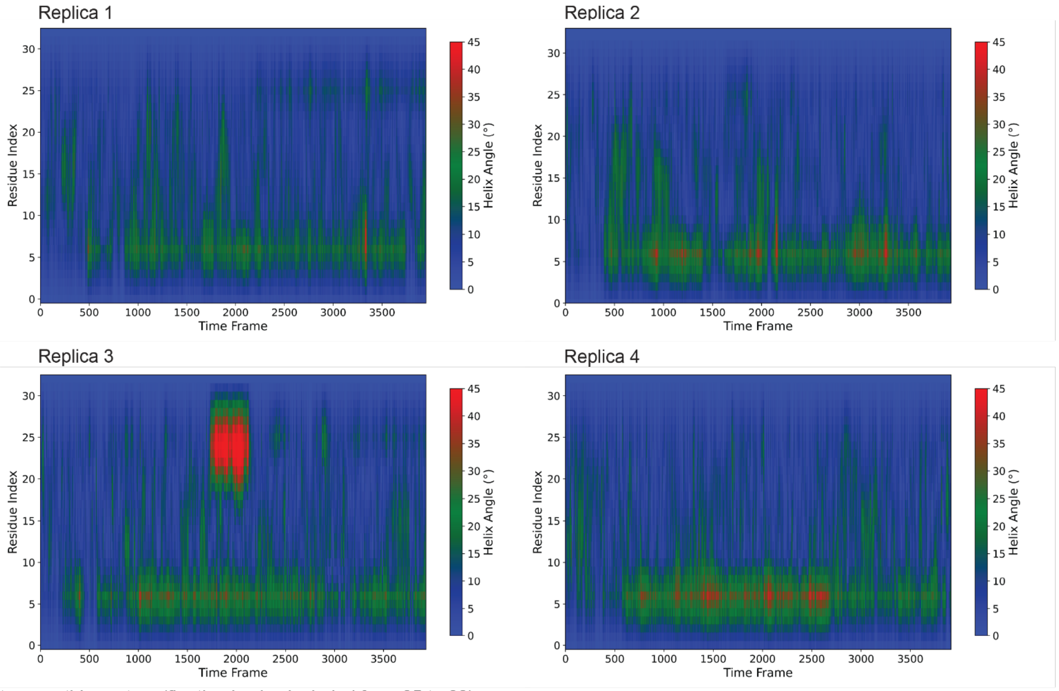


two-peptide system (floating is also included from 35 to 68)

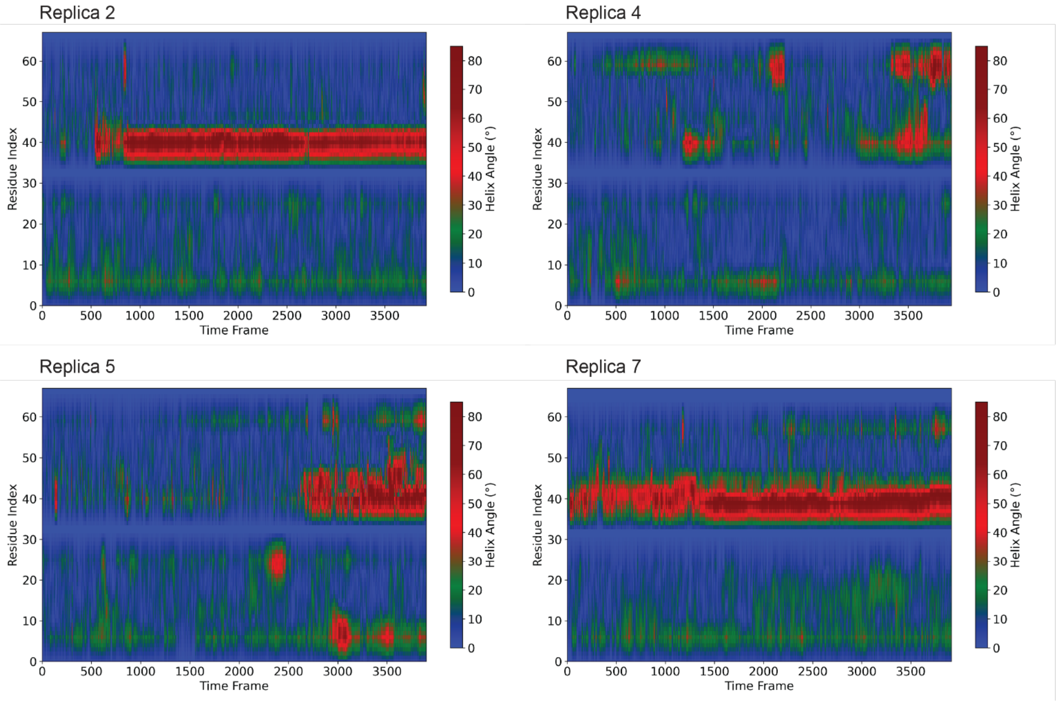


Fig B Bending angle heat map for single-bound peptide in single and multiple systems for all replicas.

We repeated the Bendix analysis for this two-peptide system (Fig B in S1 Material). The floating peptide—initially in solution—displays greater conformational flexibility, with the helix angle map

showing more pronounced bending at the N-terminal residues compared to the C-terminal end. Comparing the average bending profiles of the membrane-bound peptide between single- and two-peptide systems (Fig C in S1 Material) reveals no considerable differences aside from the slight increase in C-terminal bending. These observations suggest that the primary driver of peptide curvature is the interaction with the membrane itself, particularly the distribution of hydrophobic and hydrophilic residues along the AH and flanking regions rather than inter-peptide contacts.

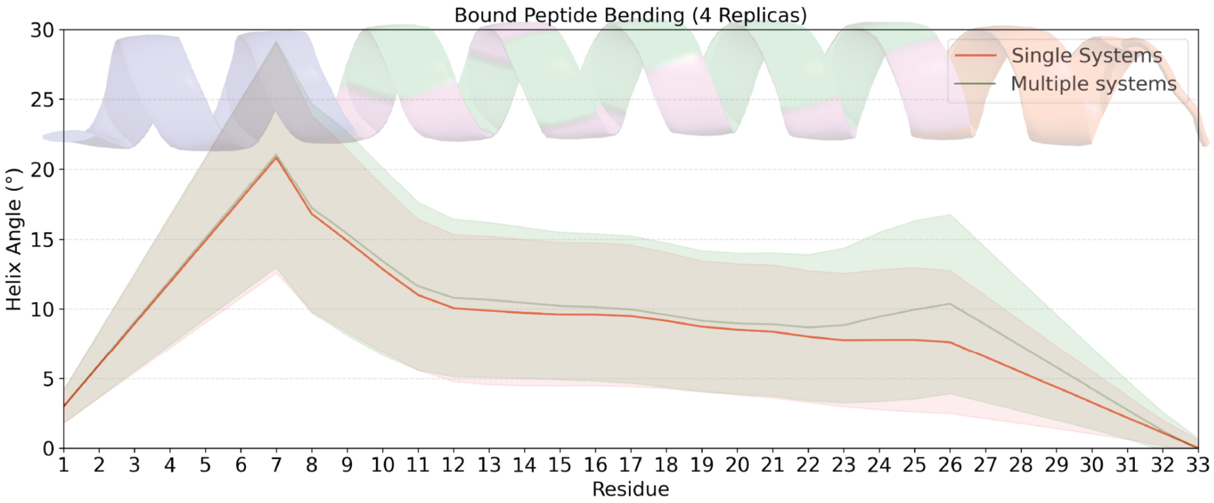


Fig C Average bending angle profile of the bound peptide over all four replicas in single- and two-peptide systems.

The highest bending is observed at the interface between the AH domain and N-terminal extension. To complement the local analysis, we also computed the global curvature of the peptide by fitting a best-fit circle to its smoothed backbone, yielding an overall radius of curvature of approximately 60 Å (Fig A in S1 Material).

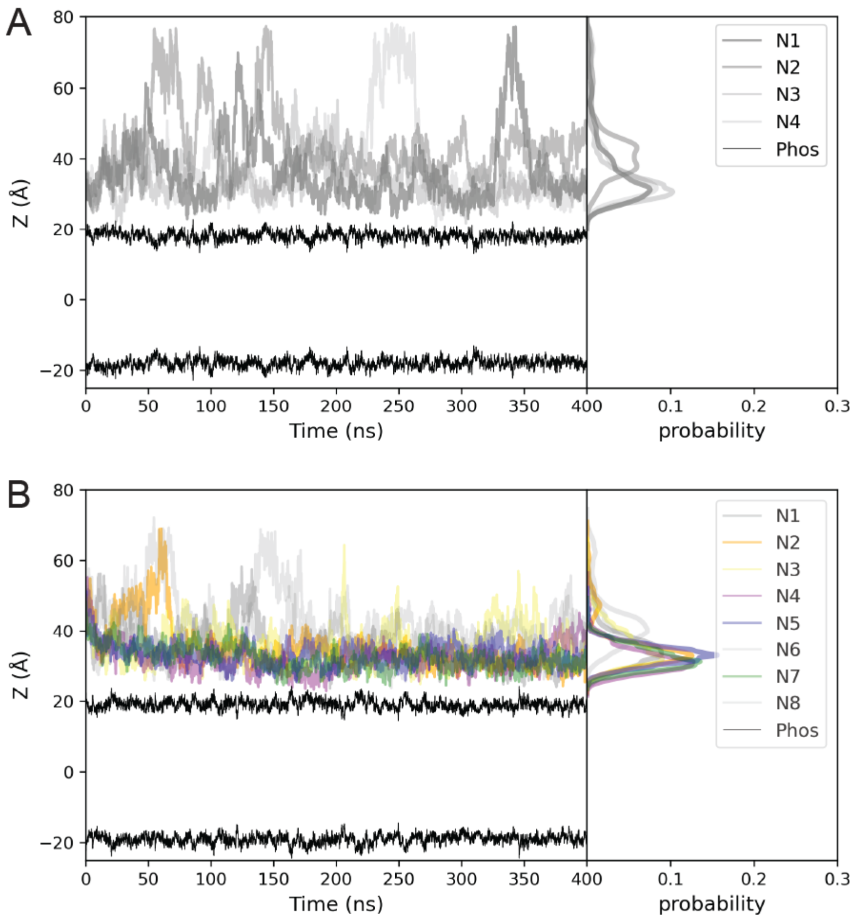


Fig D (A) Z-component of the center of mass of a single unbound peptide's AH domain. The histogram is shown on the side for each replica (n=4). (B) Z-component of the center of mass of the unbound peptide's AH domain in the presence of a bound peptide (n = 8). The histogram is shown on the side. Replicas with no interaction are colored in gray.

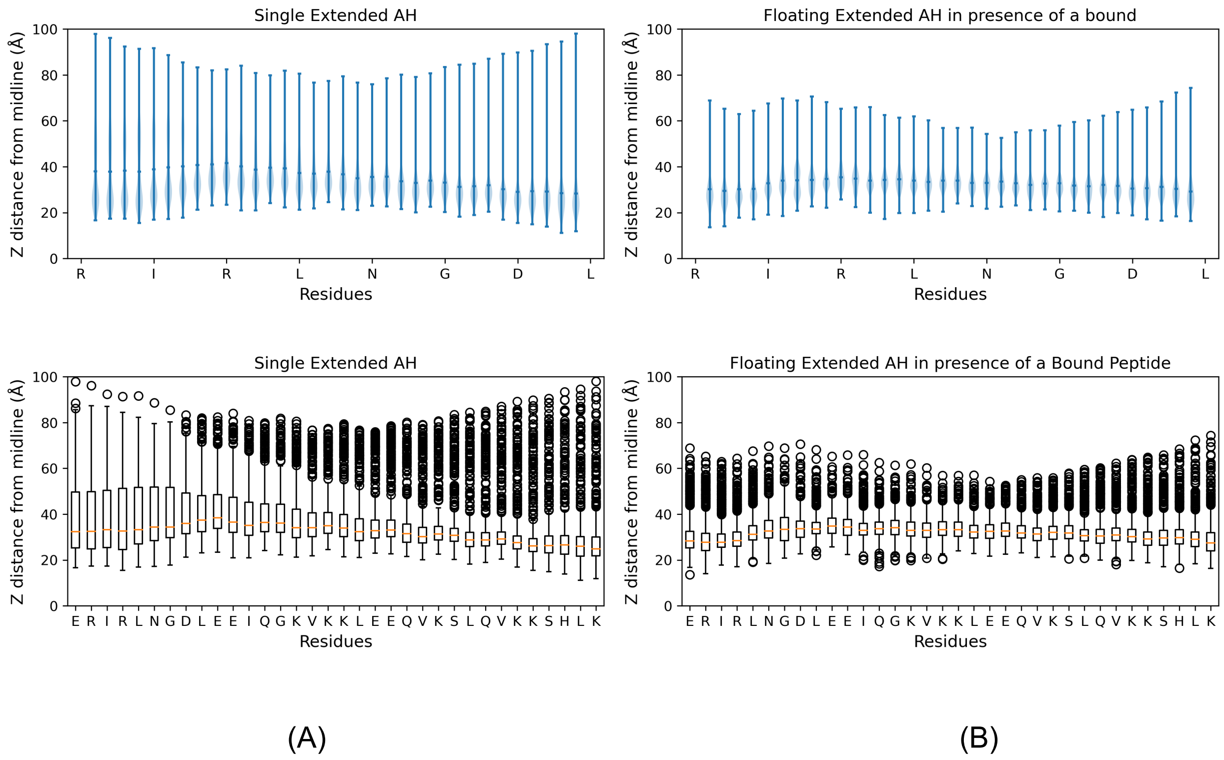


Fig E Violin and boxplot showing the median and deviation of the Z-component distance of the center of mass of the peptides from the membrane midline. (A) single unbound extended AH. (B) unbound extended AH in the presence of a bound peptide.

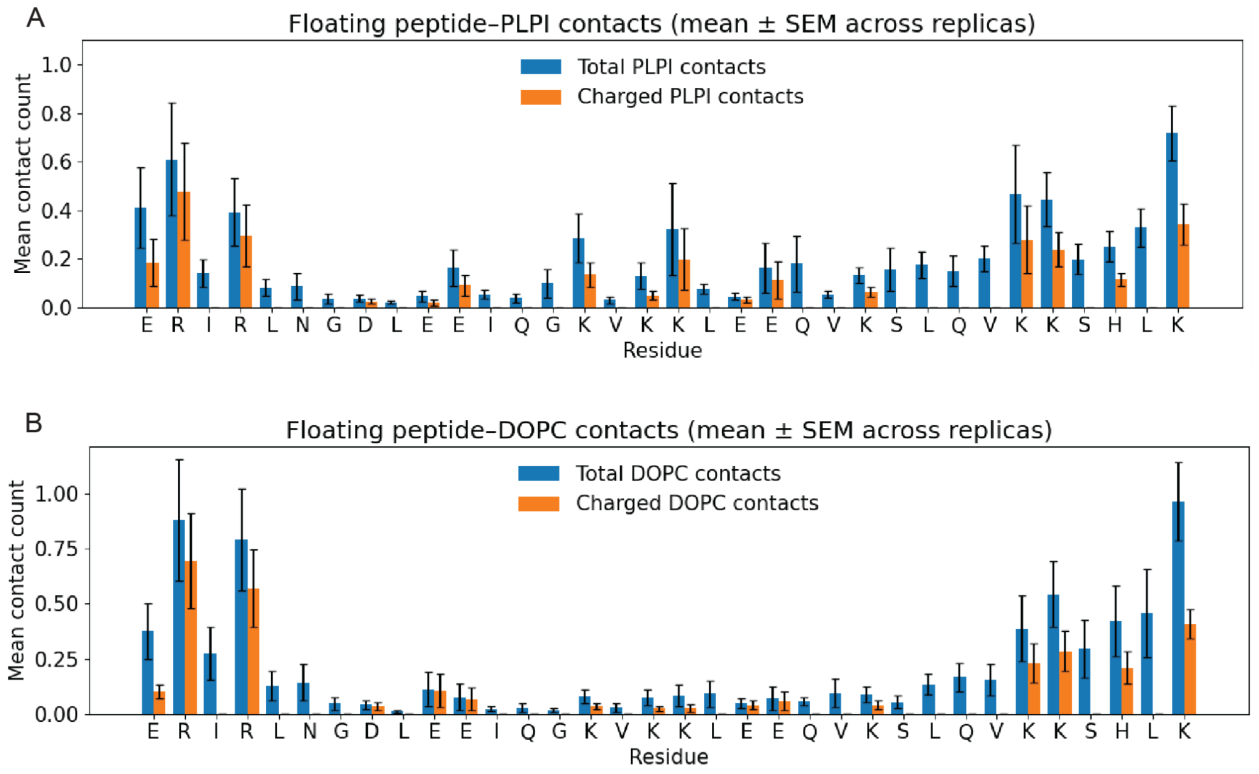


Fig F Residue-resolved lipid contacts for the floating and bound peptides. Mean contact counts per simulation frame (± SEM across eight independent replicas) between each residue of the and membrane lipids, computed using a 4.5 Å distance cutoff. Total contacts (blue) report, for each residue, the mean number of *distinct PLPI (or DOPC) headgroup residues* whose headgroup atoms (P, O2–O6, and HO2–HO6) come within 4.5 Å of any atom of that residue. Charged contacts (orange) report the mean number of *distinct PLPI (or DOPC) headgroup residues* whose charged headgroup atoms (P and O2–O6) are within 4.5 Å of charged peptide side-chain atoms (Lys NZ; Arg NE/NH1/NH2; Asp OD1/OD2; Glu OE1/OE2; and His ND1/NE2 when present). (A) floating peptide–PLPI, (B) floating peptide–DOPC.

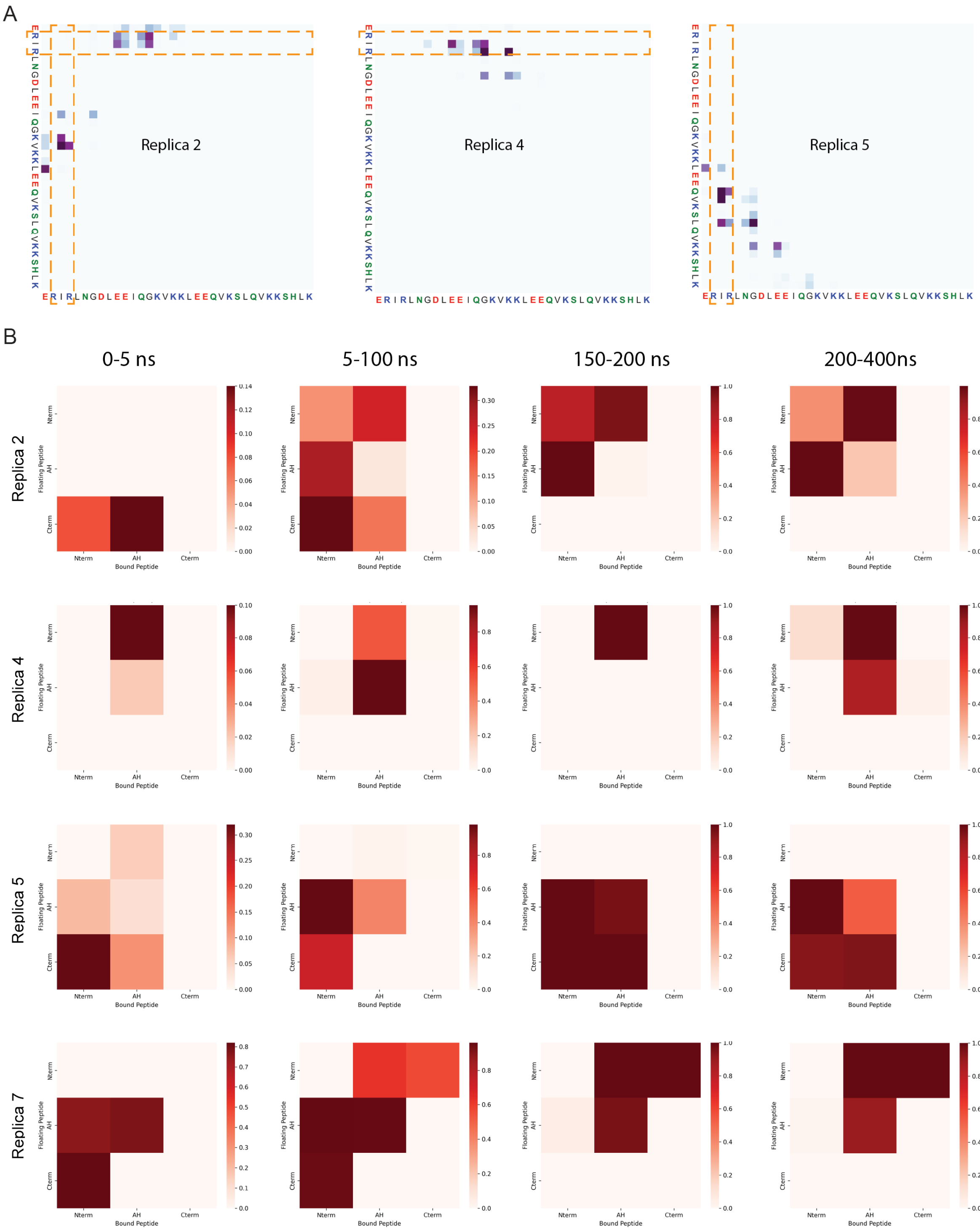


Fig G Peptide-peptide contact maps reveal how a floating and a bound AH interact. (A)The zoomed-in inter-peptide contact map for other interacting replicas highlights how charged R residues of the N-terminal form salt bridges with the other peptide. (B) The domain-based coarse-grained contact maps of interacting replicas in different time windows show two peptides interacting in an anti-parallel configuration that reaches a steady state.

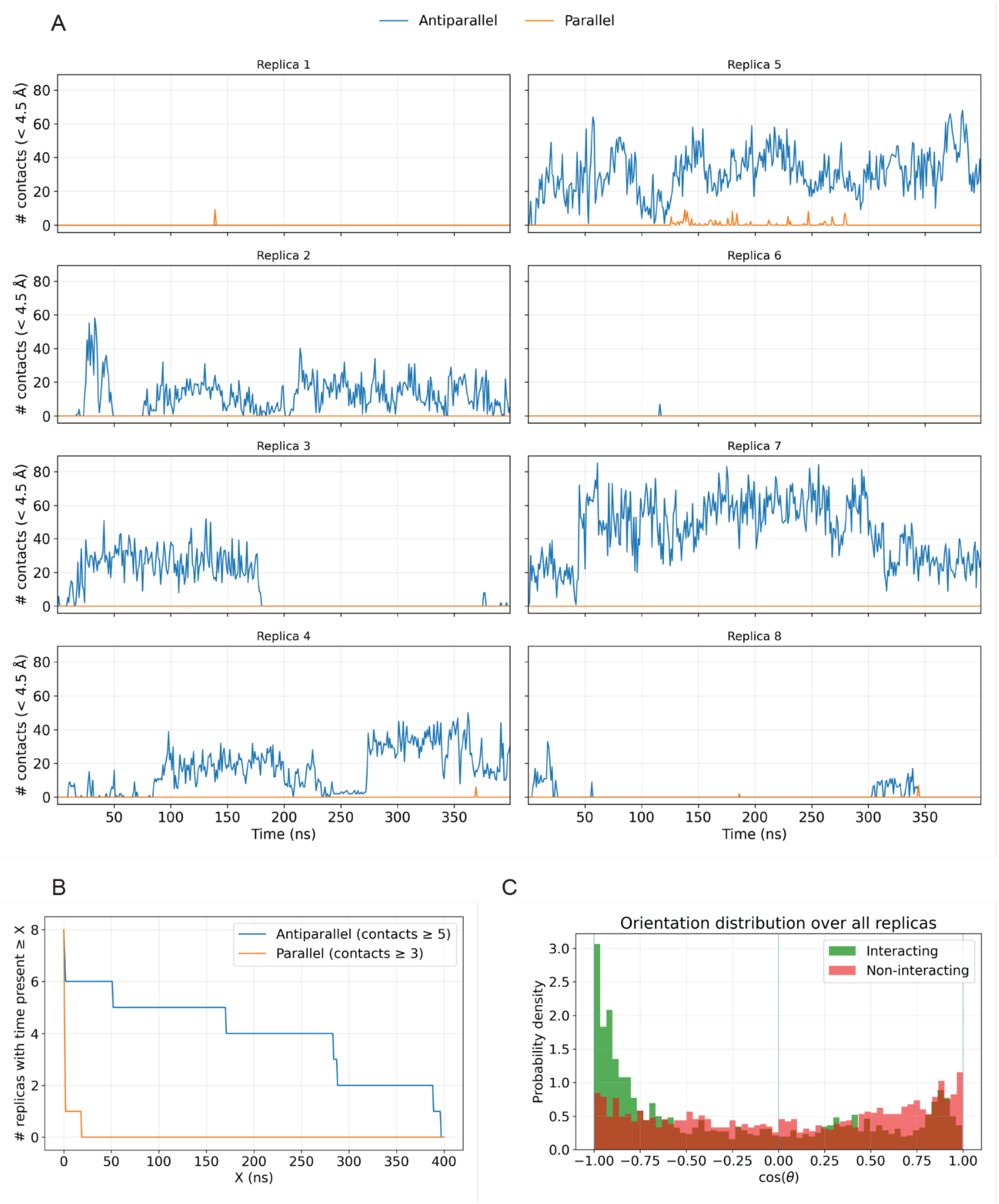


Fig H Antiparallel-biased association of a bound and a floating peptide across eight replicas. (A) Time evolution of inter-peptide contact counts (heavy-atom distance < 4.5 Å). “Antiparallel” contacts (blue) are defined as contacts between the N-terminal patch (residues 1–8) of either peptide and the second half of the other peptide (residues 17–

34). “Parallel” contacts (orange) are defined analogously using the C-terminal patch (residues 27–34) contacting the second half of the other peptide. (B) Replica persistence analysis based on cumulative time with contacts above threshold. For each replica, we computed the total time with antiparallel contacts ≥ 5 and parallel contacts ≥ 3, and plotted the number of replicas that maintain each criterion for at least X ns. (C) Distribution of peptide–peptide orientation across all replicas for interacting frames (green) and non-interacting frames (red). The orientation metric is $\cos(\theta)$, where θ is the angle between the two peptide N to C axis vectors, each defined from the COM of the N-terminal patch (residues 1–8) to the COM of the C-terminal patch (residues 27–34) using heavy atoms. Interacting frames are enriched at negative $\cos(\theta)$, consistent with an antiparallel-like bias, whereas non-interacting frames sample a broader range of orientations.

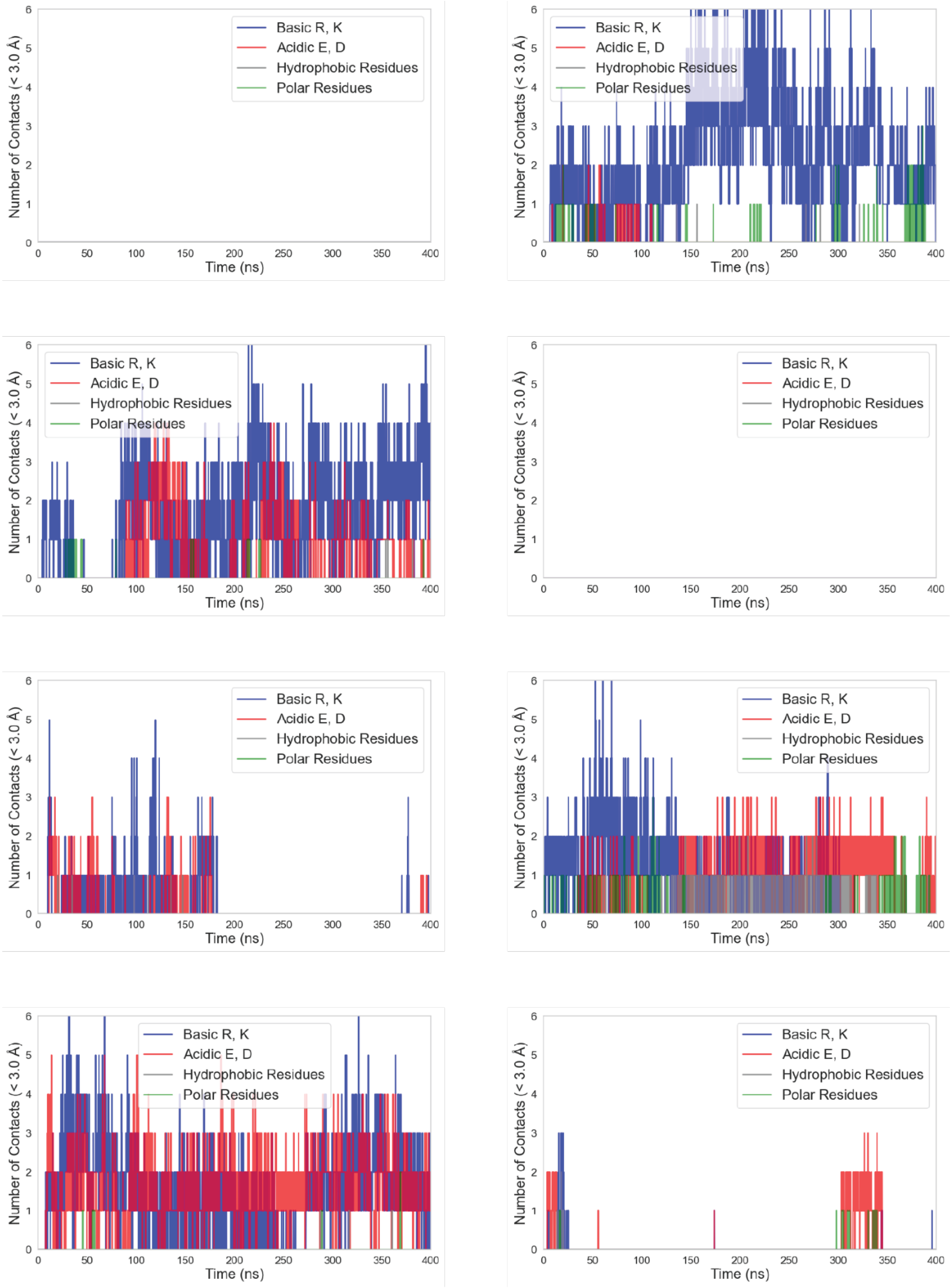


Fig I number of contacts during simulation for every 8 replicas. In replicas 1, 3, 6, and 8, peptides could not find or interact with each other. For interacting peptides (replicas 2, 4, 5, 7), the figures show how charged residues are dominant in forming contacts.

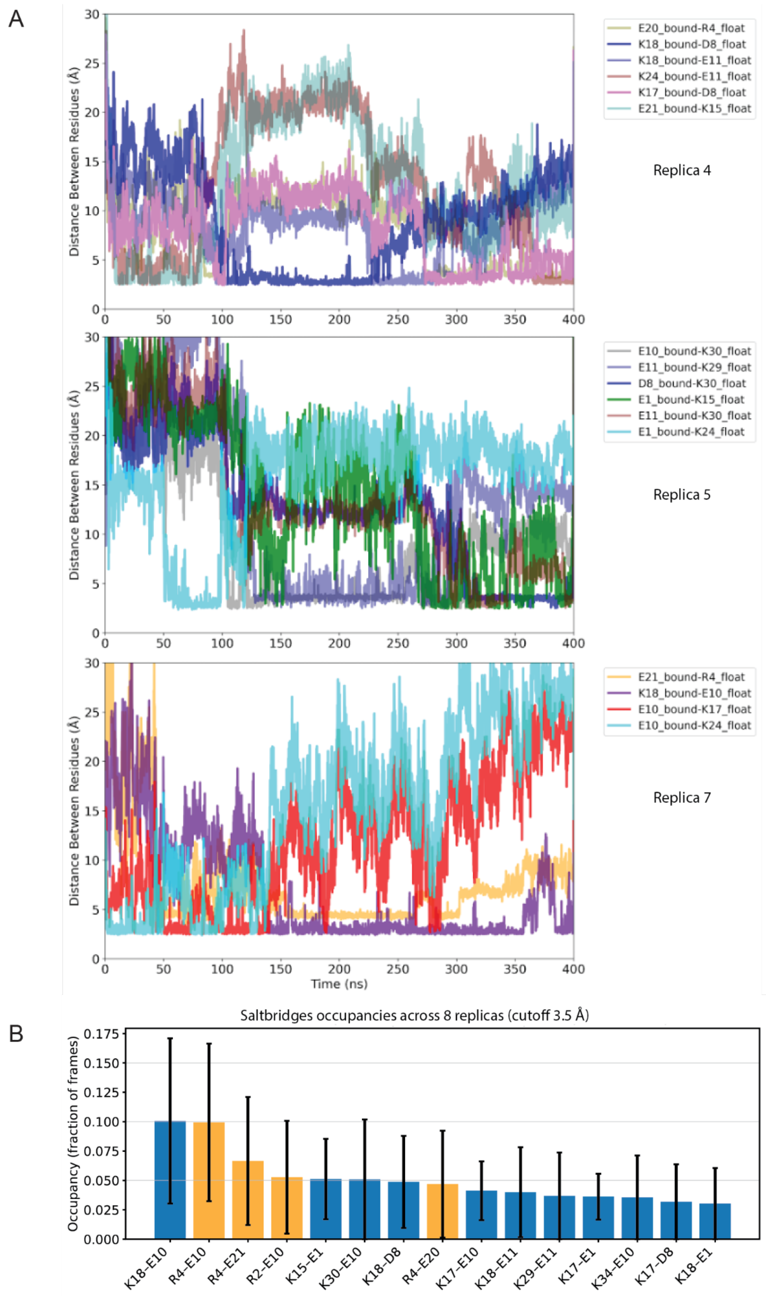


Fig J Salt-bridge dynamics between the bound and floating peptides. (A)Time evolution of residue-residuer distances for the top-ranked charged pairs using the VMD salt-bridge analysis tool for representative interacting replicas (4, 5, and 7). (B) Mean salt-bridge occupancies across all eight replicas for the most frequently observed residue pairs, computed as the fraction of analyzed frames with a cutoff distance is ≤ 3.5 Å; error bars indicate the standard deviation across replicas. The highest-occupancy contacts involve R-E residue pairs (shown in yellow).

Table A Salt-bridge occupancy across replicas for the most frequently observed inter-peptide charged residue pairs. Occupancy is reported for each replica as the fraction of analyzed frames in which a given pair satisfies the salt-bridge distance criterion (cutoff 3.5 Å), and the final column reports the mean ± standard deviation across replicas.

| Pair | Rep 1 | Rep 2 | Rep 3 | Rep 4 | Rep 5 | Rep 6 | Rep 7 | Rep 8 | Mean ± SD |
|---|---|---|---|---|---|---|---|---|---|
| **K18–E10** | 0.000 | 0.000 | 0.035 | 0.193 | 0.005 | 0.000 | 0.565 | 0.007 | 0.101 ± 0.199 |
| **R4–E10** | 0.000 | 0.487 | 0.003 | 0.305 | 0.000 | 0.000 | 0.000 | 0.000 | 0.099 ± 0.190 |
| **R4–E21** | 0.000 | 0.000 | 0.090 | 0.003 | 0.000 | 0.000 | 0.440 | 0.000 | 0.067 ± 0.154 |
| **R2–E10** | 0.000 | 0.388 | 0.003 | 0.033 | 0.000 | 0.000 | 0.000 | 0.000 | 0.053 ± 0.136 |
| **K15–E1** | 0.000 | 0.245 | 0.000 | 0.000 | 0.163 | 0.000 | 0.000 | 0.003 | 0.051 ± 0.097 |
| **K30–E10** | 0.000 | 0.000 | 0.000 | 0.000 | 0.407 | 0.000 | 0.000 | 0.000 | 0.051 ± 0.144 |
| **K18–D8** | 0.000 | 0.000 | 0.000 | 0.315 | 0.000 | 0.000 | 0.000 | 0.075 | 0.049 ± 0.111 |
| **R4–E20** | 0.000 | 0.000 | 0.000 | 0.365 | 0.010 | 0.000 | 0.000 | 0.000 | 0.047 ± 0.129 |
| **K17–E10** | 0.000 | 0.000 | 0.000 | 0.045 | 0.087 | 0.000 | 0.198 | 0.000 | 0.041 ± 0.071 |
| **K18–E11** | 0.000 | 0.000 | 0.013 | 0.307 | 0.000 | 0.000 | 0.000 | 0.000 | 0.040 ± 0.108 |
| **K29–E11** | 0.000 | 0.000 | 0.000 | 0.000 | 0.295 | 0.000 | 0.000 | 0.000 | 0.037 ± 0.104 |
| **K17–E1** | 0.000 | 0.150 | 0.037 | 0.087 | 0.015 | 0.000 | 0.000 | 0.000 | 0.036 ± 0.055 |
| **K34–E10** | 0.000 | 0.000 | 0.000 | 0.000 | 0.285 | 0.000 | 0.000 | 0.000 | 0.036 ± 0.101 |
| **K17–D8** | 0.000 | 0.000 | 0.000 | 0.255 | 0.000 | 0.000 | 0.000 | 0.000 | 0.032 ± 0.090 |
| **K18–E1** | 0.000 | 0.242 | 0.000 | 0.000 | 0.000 | 0.000 | 0.000 | 0.000 | 0.030 ± 0.086 |

Table B Salt-bridge lifetime (continuous event duration) for top pair, sorted by total time

| Pair | n_events | Total timens | Median ns | Mean ns | P95 ns |
|---|---|---|---|---|---|
| **K18–E10** | 40 | 321.0 | 2.0 | 8.03 | 41.40 |
| **R4–E10** | 50 | 280.0 | 3.0 | 5.60 | 16.65 |
| **R4–E21** | 18 | 183.0 | 1.5 | 10.17 | 36.20 |
| **K30–E10** | 19 | 162.0 | 4.0 | 8.53 | 24.70 |
| **K18–D8** | 13 | 156.0 | 4.0 | 12.00 | 45.00 |
| **R4–E20** | 5 | 146.0 | 18.0 | 29.20 | 73.80 |
| **K15–E1** | 48 | 144.0 | 2.0 | 3.00 | 6.95 |
| **R2–E10** | 43 | 142.0 | 2.0 | 3.30 | 6.90 |
| **K17–E10** | 26 | 132.0 | 2.0 | 5.08 | 17.25 |
| **K18–E11** | 22 | 127.0 | 4.0 | 5.77 | 9.00 |
| **K34–E10** | 6 | 113.0 | 19.5 | 18.83 | 33.50 |
| **K29–E11** | 23 | 111.0 | 3.0 | 4.83 | 11.80 |
| **K17–D8** | 10 | 102.0 | 5.5 | 10.20 | 32.90 |
| **K17–E1** | 27 | 98.0 | 2.0 | 3.63 | 11.90 |
| **K18–E1** | 18 | 87.0 | 3.0 | 4.83 | 16.75 |

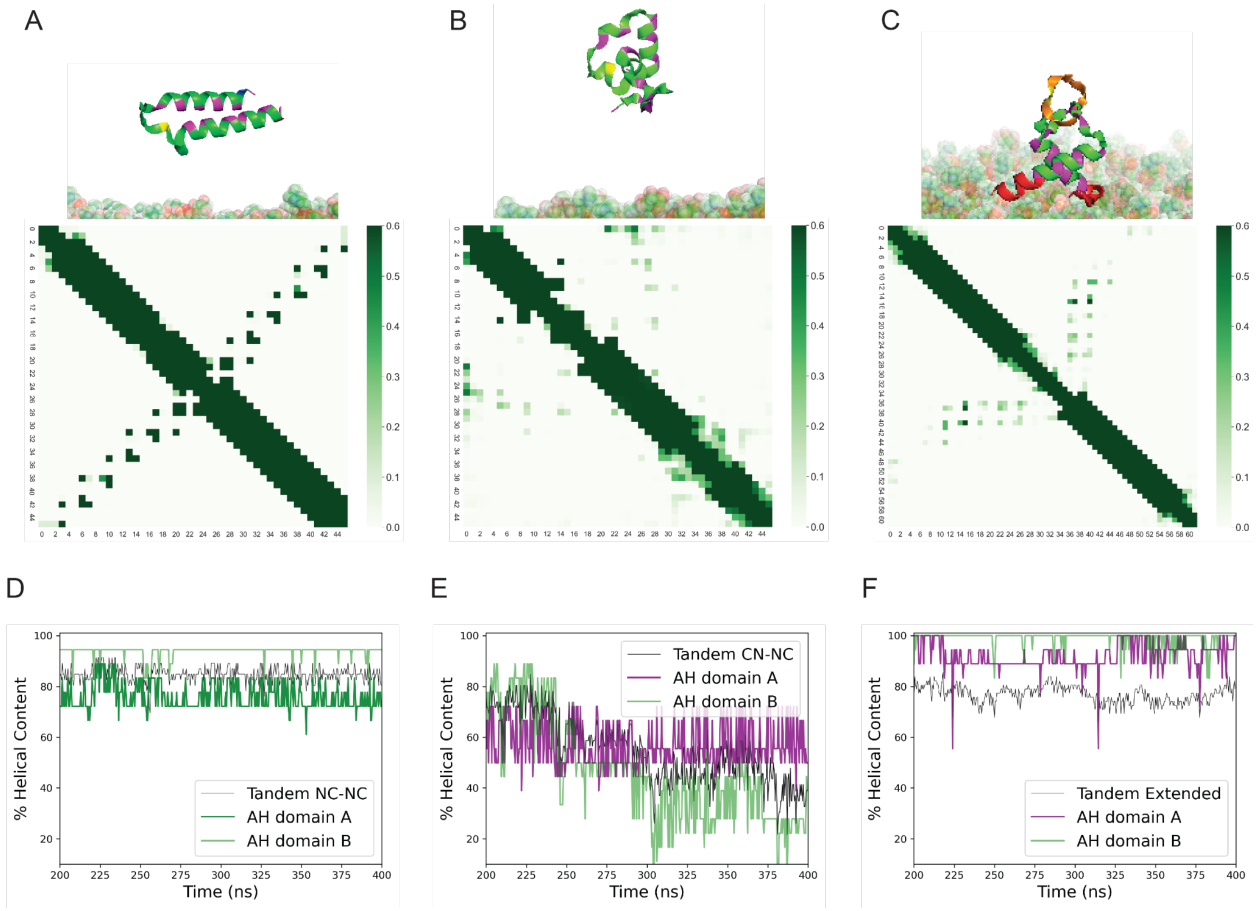


Fig K The tandem peptide remains stable when AH domains are arranged in an antiparallel configuration or extended with charged C-termini. (A) Simulation snapshots illustrate how tandem AH domains in an NC-NC arrangement hinge at the central linker, forming a stable antiparallel configuration. Sharp features in the contact map indicate strong, stable interactions between anti-parallel AH domains. (B) Tandem AH domains in a CN-NC (parallel) configuration exhibit fluctuating interactions, with the contact map showing an unstable state. (C) Extended Tandem AHs with added charged C-termini stabilize the parallel AHs in CN-NC configuration. (D-F) Helical content analysis reveals that stable states correspond to higher helical configurations. The NC configuration is shown in green, and the CN configuration is shown in purple for (D) anti-parallel, (E) parallel, and (F) extended parallel tandem AHs.

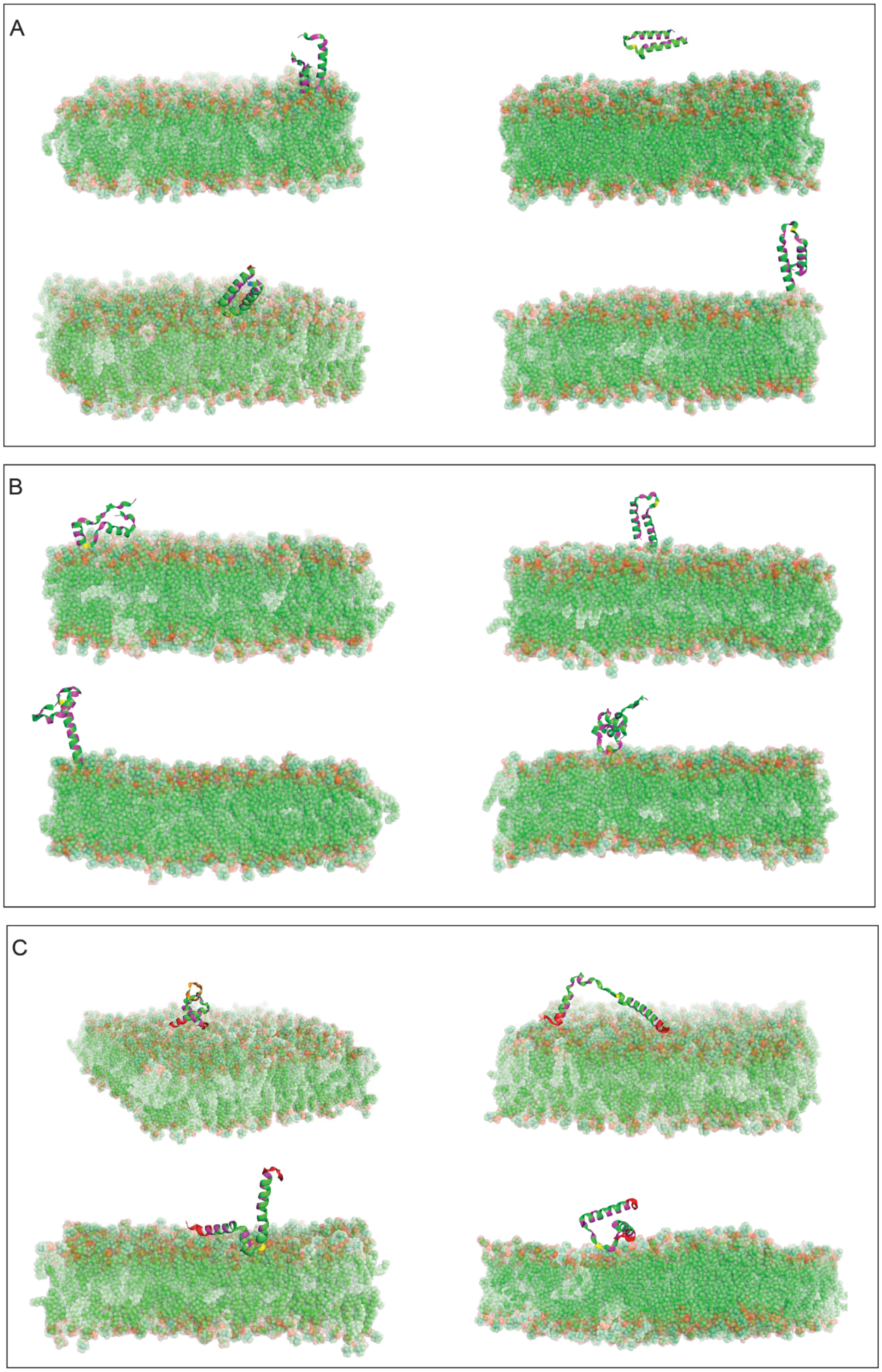


Fig L Final-frame snapshots of tandem AH simulations across independent replicates. (A) Tandem AH domains in NC-NC antiparallel arrangements, (B) Tandem AH domains in a CN-NC parallel arrangement, (C) Extended Tandem AH constructs with additional charged C-terminal domains to the parallel arrangement.

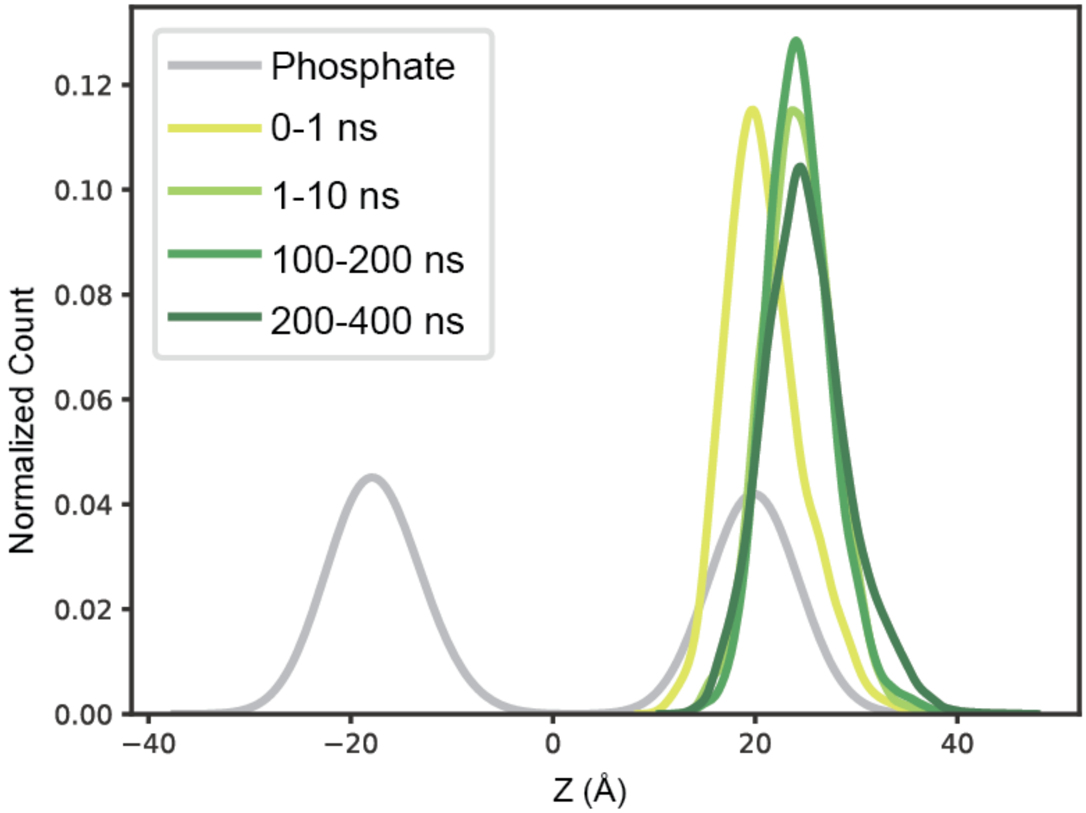


Fig M Z-component of the center of mass distribution for peptides in simulations containing two membrane-bound peptides (n = 8), shown across different time windows. As the simulation progresses, both peptides exhibit a slight upward shift, indicating gradual displacement away from the membrane interior

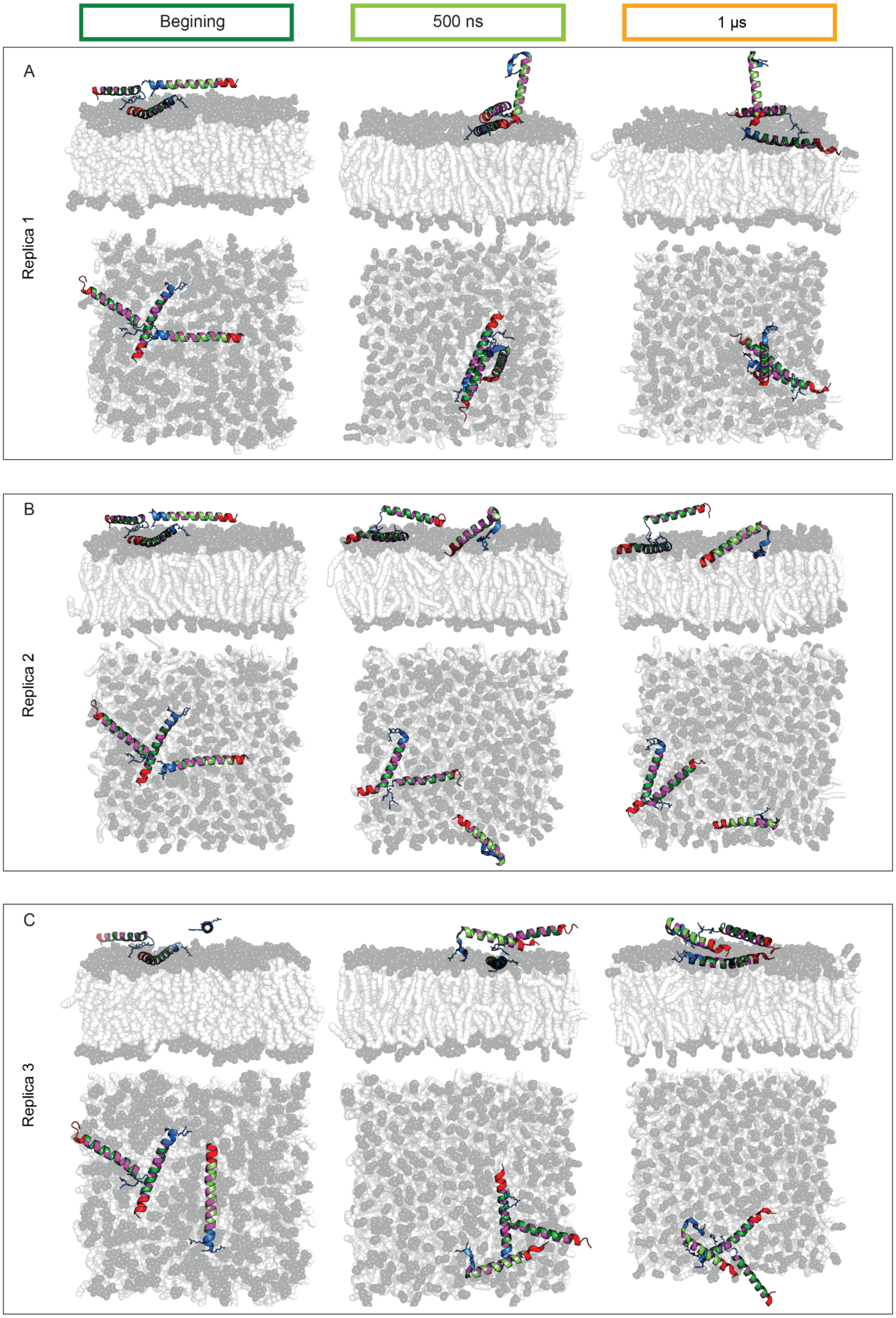


Fig N Side and top-view snapshots of simulations with three peptides at the beginning, 0.5, and 1 μs. (A) Replica 1, (B) Replica 2, (C) Replica 3.
