# Supplementary material for: Study of Protein-Protein Interactions in Septin Assembly: Multiple amphipathic helix domains cooperate in binding to the lipid membrane
Source: PLoS Comput Biol. 2026 Apr 27;22(4):e1014230. doi: 10.1371/journal.pcbi.1014230 (PMC13143081; doi:10.1371/journal.pcbi.1014230)
Supplement: Attachment — Submitted filename: Response to Reviewers.pdf [file pcbi.1014230.s003.pdf]

<u>Response to Reviewer 1:</u>

In their manuscript Mofidi et al. investigate the interactions of the amphipathic helix (AH) of Cdc12 in a lipid bilayer with molecular dynamics simulations. The experiments escalated logically from single peptide simulations to double peptide systems and other complexes. Taking the results together, the authors suggest a mechanism for how the Cdc12 of septin recruits other septins and thus starts the polymerization process. Most importantly, the authors highlight the charged residues involved in the process which could be readily validated via mutations in in vitro studies.

Overall the manuscript is written well and the figures have been presented elegantly. The following are my comments to improve the manuscript.

<u>Authors' Reply:</u> We are glad to receive Reviewer's positive evaluation of our work and appreciate their accurate summary of this study. We thank the Reviewer for their time and constructive comments on further improvement of the work.

1. I strongly recommend performing some coarse grained simulations with e.g. the Martini 3 force field to sample the peptide-peptide interactions. With the Martini force field the peptide tertiary structure has to be restrained so taking the average structure from lipid bound simulations could be a worthwhile strategy. The main message of this paper is the proposed mechanism for septin polymerization, and the four (out of eight that had binding) 400 ns simulations are very short to properly investigate the interaction. With so many degrees of freedom it is not surprising each simulation produced different and heterogeneous charge interactions. It would be highly valuable for in vitro validation to be able to point out a specific residue likely to participate in the interaction.

<u>Authors' Reply:</u> We agree with the reviewer that longer-timescale sampling would be valuable for characterizing peptide association. Following this suggestion, we tested coarse-grained Martini simulations to assess whether CG could efficiently sample peptide–peptide interactions while maintaining realistic peptide-membrane binding.

Using Martini 3 (with the standard recommendation of restraining peptide secondary structure with an elastic network model), the extended AH peptides did not adsorb to the DOPC:PLPI membrane from the solution in our setup. Similar system-dependence has been reported for peptide interactions and membrane insertion in Martini 3, where peptide-water balance and peptide-peptide association can require interaction rescaling or related corrections [1–3].

We therefore tested Martini 2, which reproduced peptide adsorption [Fig. R1], but did not yield specific peptide-peptide association. Protein-protein interaction imbalances/overaggregation are also a known limitation in Martini 2 [4], underscoring that peptide association outcomes can be highly parameter-sensitive.

Taken together, these results suggest that obtaining reliable CG sampling for this short, highly charged amphipathic helix would likely require system-specific tuning beyond the scope of the present work.

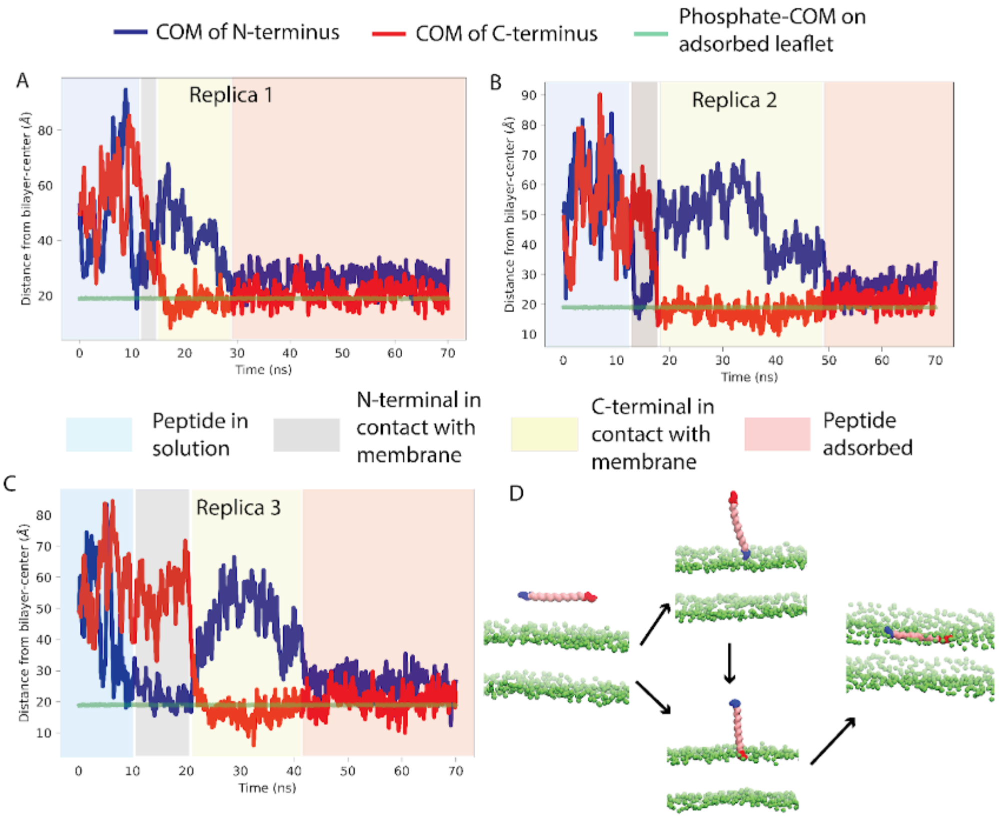

Fig. R1 - C-terminus-first adsorption of the peptide to the membrane in three independent replicas. (A-C) Distance from the bilayer center of mass as a function of time for the peptide N-terminus (blue) and C-terminus (red) centers of mass (COM). The COM of phosphate groups in the adsorbing leaflet (green) is shown as a reference for the membrane surface. Shaded regions denote the qualitative adsorption stages: peptide in solution (light blue), N-terminal contact (gray), C-terminal contact (yellow), and fully adsorbed state (pink). Across replicas, membrane association is initiated predominantly by the C-terminus, followed by stable surface-bound configurations. (D) Representative snapshots from an adsorption trajectory illustrating approach, first contact, reorientation, and final adsorbed state (N-terminus blue, C-terminus red; lipids green).

1. Thomasen FE, Skaalum T, Kumar A, Srinivasan S, Vanni S, Lindorff-Larsen K. Rescaling protein-protein interactions improves Martini 3 for flexible proteins in solution. Nat Commun. 2024;15: 6645. doi:10.1038/s41467-024-50647-9
2. Claveras Cabezudo A, Athanasiou C, Tsengenes A, Wade RC. Scaling Protein–Water Interactions in the Martini 3 Coarse-Grained Force Field to Simulate Transmembrane Helix Dimers in Different Lipid Environments. J Chem Theory Comput. 2023;19: 2109–2119. doi:10.1021/acs.jctc.2c00950
3. Spinti JK, Neiva Nunes F, Melo MN. Room for improvement in the initial martini 3 parameterization of peptide interactions. Chemical Physics Letters. 2023;819: 140436. doi:10.1016/j.cplett.2023.140436
4. Javanainen M, Martinez-Seara H, Vattulainen I. Excessive aggregation of membrane proteins in the Martini model. PLOS ONE. 2017;12: e0187936. doi:10.1371/journal.pone.0187936

2. Both ends of the extended AH domain have been neutrally capped. However, its C-terminus matches the C-terminus of full length Cdc12 and thus unless Cdc12 is capped in vitro the C-terminus would be negatively charged, reducing the net charge from +3 to +2. It is unlikely this would significantly change the attained results, but should be mentioned as a limitation.

Authors' Reply: Thank you for this helpful observation. We agree that the native C-terminus would carry a −1 charge, and thus our use of the CT2 neutral cap makes the isolated AH construct one unit more positively charged than the physiological uncapped state. We have also added a discussion stating that while this difference may modestly affect electrostatic interaction strength, we do not expect it to qualitatively alter the cooperative interaction mechanisms identified here, which are governed by conserved residue-specific salt bridges and overall charge distribution rather than by terminal charge alone. We have now noted this in the Methods that both peptide termini were capped to neutralize terminal charges, a standard approach when simulating isolated peptide fragments (revised manuscript, p. 27).

3. The use of a DOPC:PLPI 75:25 bilayer should be mentioned in the results or discussion and justified. Presumably it mimics the intracellular leaflet.

Authors' Reply: Thank you for pointing this out. We added a clear justification for the bilayer composition. In the present study, we selected a DOPC:PLPI 75:25 membrane to maintain consistency with the lipid environment used in our previous computational work (Edelmaier et al., 2025), enabling direct comparison between single-peptide and multi-peptide simulations. This lipid mixture also aligns with established experimental conditions for septin–membrane binding assays (e.g., Cannon et al., 2019; doi:10.1083/jcb.201807211). Please see Methods section on page 28.

4. Charge interactions between the peptides and the different lipid species should be investigated. Beyond hydrophobic interactions perhaps specific residues drive the binding interaction, such as the positively charged C-terminal to negatively charged PLPI.

Authors' Reply: We thank the reviewer for this suggestion. To explicitly assess charge-mediated interactions, we performed a residue-resolved contact analysis separating total lipid headgroup contacts from charged contacts between peptide side-chains and lipid headgroups. This analysis was carried out for both PLPI and DOPC lipids. The results, presented in the new Supplementary Fig. S6 (p. 42). This analysis indicates that PLPI engages not only the peptide termini but also internal residues more strongly than DOPC. We added a paragraph in the results section on p. 11 under Figure 3.

5. In figure 3A the distributions should be symmetrized to enable comparison. Presumably in one system a single peptide has migrated across the PBC to the negative side, reducing the distribution on the positive side.

Authors' Reply: Thank you for this suggestion. We considered symmetrizing the distributions but ultimately decided against it, as doing so would obscure the actual positional sampling of the peptides. Symmetrization would artificially impose bilateral symmetry that is not present in the

raw data and could give a misleading impression of the underlying dynamics, particularly in cases where a peptide genuinely crosses periodic boundaries. For clarity and to preserve the physical behavior observed in the simulations, we therefore present the unsymmetrized distributions.

6. The domain level contact maps of figure 4B should be shown for each replicate in the supplementary so that we can confirm each simulation reached a steady state. If the results are different between replicates the insufficient sampling should be mentioned as a limitation.

Authors' Reply: We thank the reviewer for this suggestion. We have now added the domain-level contact maps corresponding to Fig. 4B for each replica to Fig. S7B (p. 43). To assess whether the simulations reach a steady interaction pattern, we report these maps over successive time windows (0–5 ns, 5–100 ns, 100–200 ns, and 200–400 ns). The maps show an initial short relaxation period (0–5 ns), after which a consistent contact motif emerges and persists through the remainder of the trajectories. Across replicas, the dominant interactions are primarily mediated by the N-terminal/AH regions, with C-terminal-involving contacts generally weaker and/or transient. While the relative intensities of secondary contact blocks vary between replicas, this variability does not change the dominant contact pattern.

7. Since figure 4C is a representative example of how two peptides interact with each other, and it seems that the N-terminus of the floating peptide is unfolding, it should be mentioned as a limitation that the full length Cdc12 would influence the secondary structure and thus the interactions.

Authors' Reply: We agree with the reviewer's observation. In the context of full-length Cdc12, the N-terminal region would be structurally constrained and therefore less prone to the unfolding seen in the isolated AH construct. We now acknowledge this point as a limitation in the manuscript and note that secondary-structure stabilization provided by the full protein may influence the peptide–peptide interactions observed in our simplified model. Please see the conclusion section (p. 24).

8. Final frame snapshots of the tandem peptide systems for each replicate should be shown in the supplementary. This would enable the reader to confirm the stability per system. Figure S8 shows these for one replicate, but four replicates were performed.

Authors' Reply: We thank the reviewer for this helpful suggestion. We have now added final-frame snapshots for all four independent replicates of each tandem peptide system to the Supplementary Information. These figures (new Fig. S12) complement Fig. S11 and allow direct visual comparison across replicates, confirming the stability and reproducibility of the observed configurations (pp. 17, 50).

9. Consider publishing the full simulation trajectories in an open access repository like Zenodo.

Authors' Reply: We thank the reviewer for this suggestion. We agree that open access to the simulation data is valuable for transparency and reproducibility. We deposit the full simulation

trajectories and associated input files in an open-access repository (Zenodo), as indicated in the Data Availability section (p.33).

Minor comments

10. Consider the visualization of the Z-COM boxplots. Currently the large amount of outliers merge all together taking focus away from the main message. Maybe omit the outliers or make them smaller. In Fig 3 B and C the Y-axis could be increased so that the distribution of early residues is visible. Additionally the grey line in the boxplots is not explained in the legend, presumably it is the phosphate peak.

Authors' Reply: Thank you for these helpful suggestions. In the revised figures, we have adjusted the y-axis range in Fig. 3B and 3C (p. 10) to make the distribution of the early residues more visible. We also modified the styling of the boxplots to reduce visual clutter by removing the outliers. In addition, we now explicitly describe the meaning of the grey reference line in the figure legend.

12. To improve readability for a novice in the field the colour of lipids in the renders could be standardized. In figures 1 and 2 they are dark blue and cyan, in figure 3 red and grey, in figures 4 and 6 orange and white, and in figure 5 black and white.

Authors' Reply: We thank the reviewer for this suggestion. In the manuscript, peptide coloring is kept consistent across all figures to emphasize the amphipathic helix, which is the primary focus of this study. Lipid colors are intentionally varied between figures to provide visual contrast and to distinguish different simulation setups; for example, tandem peptide systems are shown using a green lipid color scheme, single-peptide systems in blue, bound–unbound peptide systems in red and gray, and two-bound-peptide systems in dark and light gray. In all cases, lipid coloring is used for contextual visualization and does not encode chemical identity or quantitative information.

13. In figures 4 and 6 the phospholipid tails have been rendered in some low resolution surface representation making them messy.

Authors' Reply: We thank the reviewer for this helpful comment. We have revised Fig. 4 by cropping the lipid tails and adjusting the view to reduce visual clutter and more clearly highlight the relevant peptide–peptide interactions. The phospholipid tails in Figs. 4 and 6 were intentionally rendered using surface representations to solely illustrate membrane position. Whereas peptides were rendered in higher detail to emphasize their structure and peptide–peptide interactions.

14. In figure 3A there is a typo in the legend for the green line.

Authors' Reply: Thank you for catching this. We have corrected the typo in the Figure 3A legend in the revised manuscript.

15. In figure 5C there is a typo in the legend for the dark green line.

Authors' Reply: Thank you for pointing this out. We have corrected the typo in the Figure 5C legend in the revised manuscript.

Response to Reviewer 2:

The authors report molecular dynamics simulation study of how single and paired extended amphipathic helix (AH) domain of septins interact with lipid bilayer membranes and with each other.
The novelty of the paper lies in providing molecular level insights into the orientation of the AH domain on the membrane surface and the discovery that two AH domains prefer to interact via an anti-parallel arrangment on the membrane surface, providing valuable insights into septin higher-order assembly into filaments or other structures important for various cellular functions.

Overall the paper is well organized and clearly written.
I like the paper as it is interesting to learn that AH domain interaction with each other could be important in driving oligomer assembly on the membrane surface.
I would recommend publication after addressing the following issues.

Authors' Reply: We appreciate the Reviewer's positive view and accurate summary of our work. We are grateful for their constructive comments on several aspects of the study, which we carefully took into account during the revision.

1. The authors claimed that a curved membrane may better accommodate multiple AH domains (Abstract and Page 18). It is unclear how this claim is supported by the results, which showed that the binding of multiple peptides induce lipid packing defects (increase in area/lipid and
decrease in lipid order parameter) but do not show stronger binding to regions of lipid packing defects.
Please provide some clarification on this.

Authors' Reply: Thank you for this important point. We agree that our original wording could be interpreted as implying a direct preference of AH domains for pre-existing lipid packing defects, which we did not explicitly demonstrate. Our intent was instead to suggest that the collective insertion of multiple AHs generates local membrane deformations and packing defects, which are more readily accommodated by curved membranes than by flat bilayers. We have clarified this distinction in the Abstract and Discussion by reframing the claim to emphasize membrane adaptability rather than preferential binding to defects (pp. 1-2, 22, 25-26).

2. The authors showed salt-bridge formation in contact map from Replica 7 for the system with bound + floating AH (Figure 4).

Is this or similar salt-bridges also exist from the contact maps for the other replicas (2, 4, 5 from Figure S6)?
Please show the contact maps for the other replicas for completeness.

Authors' Reply: We thank the reviewer for this suggestion. We have now added contact maps for the additional interacting replicas (replicas 2, 4, and 5) to the Supplementary Information (Fig. S7). These contact maps show interaction patterns consistent with those observed in Replica 7, including salt-bridge formation between charged arginine residues in the N-terminal region and acidic residues on the other peptide (pp. 12, 43).

3. The authors showed that when two peptides in parallel arrangement are embedded inside the membrane, in 75% of the cases the peptides remain bound to the membrane and interact with each other but in 25% of the cases one of the pair may become dissociated from the membrane (Page 15-16 and Figure 5B).

As the parallel arrangement was shown to be less stable than the anti-parallel one, please explain what is the rationale for using the parallel one?
Why not place the two peptides in the anti-parallel arrangement?

Authors' Reply: Thank you for this question. We deliberately chose the parallel arrangement as a conservative test case, as it represents a less stable interaction geometry compared to the anti-parallel configuration. Our goal was to assess whether peptide–peptide interactions remain sufficiently strong to promote cooperative behavior even under these less unfavorable conditions. The observation of partial membrane dissociation of one peptide in 25% of cases demonstrates the robustness of AH–AH interactions. We have now clarified this rationale in the revised manuscript. In addition, we note that both our atomistic work and supporting coarse-grained simulations favor anti-parallel arrangements, which we now reference to provide a broader context for the proposed assembly mechanism (pp. 17-18).

4. What is the effect of having more than two peptides on the inter-peptide interactions?
Specifically, how would an additional peptide bind to a peptide pair in anti-parallel fashion?
In anti-parallel or parallel fashion?
Some additional simulations to address this would be useful.

Authors' Reply: We thank the reviewer for raising this important question. In response, and in conjunction with a related comment from another reviewer, we have performed new simulations containing three amphipathic helix (AH) peptides. In these simulations, a third peptide was introduced into systems where a bound peptide and a second peptide had already formed a stable antiparallel interaction at the membrane interface.

Across multiple replicas, the third peptide adopted antiparallel orientations and stable interactions that are compatible with the salt-bridge motifs identified in the two-peptide systems. In addition, we observed enhanced membrane engagement of the third peptide in the presence of pre-assembled AHs, including C-terminal insertion in replica 2, events not observed in single-peptide simulations. Representative snapshots from these simulations are provided in the

Supplementary Information (Fig. S14). Together, these new simulations provide direct support that the antiparallel AH interaction motif can extend beyond a single peptide pair in small multivalent systems (pp.20, 21, 30, 52).

5: Are there examples of other AH domains that also form anti-parallel arrangement stabilized by electrostatic interactions? Is anti-parallel AH domains a general motif that facilitates higher order assemblies? Some discussion on this would be helpful.

Authors' Reply: We thank the reviewer for this insightful question. We have expanded the Discussion to provide examples from other lipid-associated assemblies. In particular, we now cite work on exchangeable apolipoproteins, where antiparallel amphipathic helix packing has been proposed and salt-bridge networks have been implicated in stabilizing preferred registries of antiparallel helix association. While the specific residue-level interactions identified here are septin-specific, we clarify that antiparallel AH–AH association stabilized by electrostatics may represent a recurring, though not universal, organizational principle for cooperative membrane-associated assemblies (p. 23).

Minor issues/comments:

1. In the first Results section the authors mentioned that simulations of isolated AH segment (without flanking regions) do not show significant curvature (Page 6 Line 8). Please cite prior simulation work (Edelmaier et al. as mentioned on lines 3-4 on Page 8?) or data.

Authors' Reply: Thank you for pointing that out. The citation has been added accordingly.

Figures:
2. Figure 2D legend: "Helix" angle should have been "Bending" angle instead?

Authors' Reply: We thank the reviewer for pointing this out. The angle shown in Fig. 2D (and Fig. 2E) was computed using the Bendix plugin in VMD and quantifies the local bending of the helical axis. We have corrected the figure legend and labels to read "local helix bending angle" to avoid ambiguity (pp. 7-8).

3. Figure 3A plot legend: peptide is misspelled as "peptied" for the green curve.

Authors' Reply: Thank you for catching this. We have corrected the typo in the Figure 3A legend in the revised manuscript.

4. Figure 2E: The authors labeled the x-axis using Time Frame. It might be better to convert that to simulation time or state how often each frame was saved (it seems to be a frame per 0.1 ns as the total simulation time is 400 ns (see Table 1)).

Authors' Reply: We agree and thank the reviewer for this suggestion. We have revised Fig. 2E to display simulation time in nanoseconds rather than frame index.

5. Figure 3B and C: What are the black circles? The actual data points that contribute to the box plot showing mean values? Please explain.

<u>Authors' Reply:</u> Thank you for pointing out this ambiguity. In the original version of Fig. 3B and 3C, the black circles represented outliers (fliers) in the boxplots, defined as data points lying beyond 1.5 times the interquartile range. In the revised figures, these outliers have been omitted from the boxplots for clarity, as they compress the central distributions and obscure residue-specific trends. These rare events do not affect the median or interquartile ranges. The boxplots now display the median and interquartile range only, and the figure legend has been updated accordingly.

6. Figue 4C: It is somewhat difficult to identify the charged resides forming the salt-bridges from the simulation snapshot. Perhaps the authors can include a more detailed zoomed-in view of
a few of the salt-bridges here?

<u>Authors' Reply:</u> We thank the reviewer for this helpful suggestion. We have revised Fig. 4C by increasing the zoom level and adding arrows to explicitly indicate representative charged residues involved in salt-bridge interactions. These annotations make the interacting residues clearly identifiable

7. Figure 4 legend for panel (D): The "n" in "number" should be capitalized.

<u>Authors' Reply:</u> We thank the reviewer for pointing this out. We have checked the Figure 4D legend and confirm that "Number" is capitalized in the revised manuscript.

8. Figure 6 legend for panel (B): The authors wrote "charged residues that are detrimental to forming salt-bridges".
Do they mean "instrumental" instead of "detrimental"?

<u>Authors' Reply:</u> Thank you for pointing out this wording issue. Our intended meaning was "determining," not "detrimental." We appreciate the suggestion and have revised the figure legend accordingly.

Materials and methods:

9. It is not clear as to the choice of the lipid composition for the lipid bilayer.
Please explain why 75:25 DOPC:PLPI is used?

Although this seems to follow what some of the co-authors used in a prior publication (Edelmaier et al. Biophysical Journal, 2025), some explanation/justification would still be helpful to the readers as to understand why this particular composition is representative.

<u>Authors' Reply:</u> Thank you for pointing this out. We added a clear justification for the bilayer composition. In the present study, we selected a DOPC:PLPI 75:25 membrane to maintain consistency with the lipid environment used in our previous computational work (Edelmaier et

al., 2025), enabling direct comparison between single-peptide and multi-peptide simulations. This lipid mixture also aligns with established experimental conditions for septin–membrane binding assays (e.g., Cannon et al., 2019; doi:10.1083/jcb.201807211). Please see page 28 under lipid bilayer.

Response to Reviewer 3:

This manuscript uses all-atom molecular dynamics simulations to investigate how extended amphipathic helix (AH) domains of Cdc12 interact with lipid bilayers, how multiple AHs form peptide–peptide contacts (notably via salt bridges), and how these interactions may contribute to septin curvature sensing and cooperative assembly. The authors analyze single and double AH constructs (including tandems) in the presence of planar DOPC:PLPI bilayers and derive a mechanistic model for septin membrane recruitment and filament assembly. The work is potentially interesting and relevant to readers of PLOS Computational Biology, particularly those interested in membrane–protein interactions and cytoskeletal assembly. However, in its current form the manuscript over-interprets results obtained under relatively restricted simulation conditions, and several key analyses, controls, and clarifications are missing. I therefore recommend Major Revision before the manuscript can be considered for publication.

Authors' Reply: We appreciate the acknowledgment of the potential interest and relevance of our work to the study of membrane–protein interactions and septin assembly. We acknowledge the reviewer's concerns regarding possible over-interpretation of our results under the current simulation conditions and agree that additional clarification and expanded analyses will strengthen the manuscript. We are grateful for their constructive comments on several aspects of the study, which we carefully took into account during the revision.

1. The central narrative of the paper is that multiple AH domains "cooperate" in a way that is particularly suited to curved membranes, and the Discussion explicitly proposes a mechanism of septin polymerization on curved bilayers. However, all simulations appear to have been carried out on planar bilayers of a single composition (DOPC:PLPI 75:25). The conclusion that curved membranes "may better accommodate multiple AH domains" is therefore largely inferential. To support such a strong mechanistic statement, the authors should either:
(a) perform additional simulations on explicitly curved membranes (e.g., cylindrical or vesicular geometries, or at least buckled bilayers), and quantify how AH binding, peptide curvature, and lipid defects differ from the planar case; or
(b) substantially temper the claims throughout the manuscript, clearly labeling them as hypotheses rather than conclusions, and revising the title/abstract to avoid overstatement.
At present the text reads as if a curvature-sensing mechanism has been demonstrated, which is not fully supported by the presented data.

Authors' Reply: We thank the reviewer for highlighting this important point. We agree that our simulations were performed exclusively on planar bilayers and therefore do not directly demonstrate curvature sensing or preferential binding to curved membranes. We have revised the manuscript to explicitly clarify this limitation. We now frame membrane curvature as a

biophysical context in which the cooperative AH–lipid interaction mechanisms identified here are likely to be enhanced, rather than as a demonstrated outcome of the simulations.

While we believe that explicit simulations on curved membranes would be a valuable future direction, such calculations are beyond the scope of the present study. The current work instead focuses on defining the molecular determinants of cooperative AH binding on planar membranes, which we propose provide a mechanistic basis for curvature sensitivity observed experimentally.

The Discussion has been revised to clearly label curvature-dependent interpretations as hypotheses, and the abstract has been adjusted to avoid overstatement (pp. 1-2, 25- 26).

2.Each system is simulated for 400 ns with 4–8 replicas, but the manuscript provides relatively little information about convergence diagnostics, especially for key observables such as peptide curvature, salt-bridge occupancy, peptide–membrane residence times, and lipid order parameters.
• The authors should quantify and report time evolution and replica-to-replica variability for central metrics (e.g., radius of curvature, inter-peptide contact probabilities, lipid order/disorder near vs. far from the peptides).
• It would be helpful to show that the major structural states (e.g., anti-parallel vs. parallel arrangements) are robust to initial conditions and not dependent on a particular starting geometry or transient fluctuation.
• For the "dissociation" of one peptide in the two-bound system (25% of replicas), more rigorous analysis is needed to demonstrate that this is not simply a finite-time fluctuation within 400 ns.
Without such convergence and uncertainty analysis, it is difficult to judge how reliable the inferred "preferred" configurations and mechanistic inferences are.

Authors' Reply: We thank the reviewer for raising this important point regarding convergence diagnostics and robustness across replicas. We now quantify the time evolution of inter-peptide contacts in each replica and summarize replica-to-replica variability using persistence analysis (Fig. S8–B). To assess robustness of the dominant arrangement, we operationally define antiparallel-like and parallel-like association based on which peptide terminus mediates contacts with the partner (N-terminal patch vs. C-terminal patch contacting the second half of the other peptide) and evaluate these metrics across all replicas. The results show that N-terminal-mediated contacts are consistently more frequent and longer-lived than C-terminal-mediated contacts, indicating that the predominant association mode is reproducible across independent replicas rather than arising from a single trajectory or transient event. We further added an orientation analysis comparing the distribution of relative peptide alignment during interacting versus non-interacting frames, showing that interacting frames are enriched in negative cos(θ), consistent with an antiparallel-like bias (Fig. S8C).

In addition, to address convergence and variability in a key mechanistic observable, we expanded our salt-bridge analysis. We provide time evolution traces of representative charged residue–pair distances (Fig. 4E and Fig. S10A), and we now quantify salt-bridge occupancies across replicas (Table S1; Fig. S10B) as well as dwell-time statistics (lifetimes) for the same residue pairs (Table S2). These additions explicitly report both time evolution and replica-to-replica variability for charged interactions implicated in AH–AH association.

Together with the contact maps already apparent in Fig. 4A and Fig. S7, these analyses provide replica-to-replica characterization of inter-peptide organization and its dominant structural state. We note that each replica was initiated from identical starting coordinates but with different randomized initial velocities, which is a standard approach for generating statistically independent MD replicas.

With respect to the partial dissociation observed in a subset of replicas (25%) in the two-bound peptide system, we have revised the text to interpret this outcome conservatively as a trajectory-level event within the 400 ns sampling window, rather than as evidence of a converged dissociation probability or a distinct equilibrium state. We emphasize that most replicas maintain membrane association over the simulated timescale, but that occasional partial detachment can occur in the presence of a second peptide, consistent with competition between peptide–peptide and peptide–membrane interactions. We now explicitly note that longer trajectories and/or enhanced sampling would be required to quantify state lifetimes and free-energy differences associated with this behavior.

Please see revised manuscript pp. 14-16, 42, 44, 45, 47-48.

3. The membrane patches are approximately 11 × 11 nm² with two AH peptides and relatively high local peptide density. This raises concerns that some of the observed effects—particularly the "upward shift" of peptide centers of mass and changes in APL and lipid order parameter maps—might be influenced by the small system size and periodic boundary conditions rather than generic membrane responses.

- Please justify the chosen membrane dimensions in light of the peptide size and number, and discuss possible box-size artifacts (e.g., peptide–peptide interactions through periodic images).
- Consider running at least one larger-patch control (or provide evidence from prior work) to demonstrate that the qualitative conclusions about lipid packing defects and peptide displacement are robust to box size.

<u>Authors' Reply:</u> We thank the reviewer for raising this important point. The membrane dimensions were deliberately chosen to capture local peptide–lipid and peptide–peptide interactions at all-atom resolution while maintaining computational feasibility. The extended AH peptides span ~4–5 nm, leaving several nanometers of intervening lipid between peptides and their periodic images. Lipid perturbations induced by amphipathic helices are known to be highly localized, typically decaying within 1–2 nm from the insertion site, suggesting that the present system size is sufficient to resolve local membrane reorganization.

We acknowledge that periodic boundary conditions may influence the absolute magnitude of membrane deformation or lipid order parameters. However, our conclusions are based on relative comparisons between single- and dual-peptide systems within the same simulation box, for which qualitative trends—such as localized packing defects, asymmetric lipid disorder, and modest peptide displacement—remain robust. We have clarified these points in the method section and explicitly discussed finite-size effects as a limitation. While larger-patch simulations would be valuable, they are beyond the scope of the present study (p. 27).

4.The mechanistic model extrapolates from systems containing at most two AH peptides (or tandem constructs) to septin filaments and higher-order assemblies. Yet no simulations of longer arrays of AH domains or full septin oligomers are presented.
• The authors should more clearly delineate which conclusions are directly supported by their simulations (two peptides / tandem constructs) and which are speculative extrapolations to filament-scale behavior.
• If possible within reasonable computational cost, it would strengthen the manuscript to include at least one simulation containing a short linear array of three or more AHs to test whether the proposed anti-parallel salt-bridge motif can be propagated along a putative filament.
At minimum, the language in the Abstract, Author Summary, and Discussion should be moderated to avoid implying that filament assembly mechanisms have been directly observed.

Authors' Reply: We thank the reviewer for raising this important point regarding extrapolation from small systems to higher-order septin assemblies. In response, we have taken two complementary steps.

First, we have moderated the language throughout the Abstract, Author Summary, and Discussion to clearly distinguish conclusions that are directly supported by simulations of two AH peptides or tandem constructs from more speculative extrapolations to filament-scale behavior.

Second, to directly test whether the proposed antiparallel salt-bridge motif can be propagated beyond a single peptide pair, we performed new simulations containing three independent AH peptides. In these simulations, a third peptide was introduced into systems where a bound peptide and a second peptide had already formed a stable antiparallel interaction. Across multiple replicas, the third peptide adopted antiparallel orientations and stable interactions that are compatible with the salt-bridge motifs identified in the two-peptide systems. In addition, we observed enhanced membrane engagement of the third peptide in the presence of pre-assembled AHs, including C-terminal insertion in replica 2, events not observed in single-peptide simulations. Representative snapshots from these simulations are provided in the Supplementary Information (Fig. S13). Together, these new simulations provide direct support that the antiparallel AH interaction motif can extend beyond a single peptide pair in small multivalent systems (pp. 2, 20, 21, 30, 52).

5. Salt bridges between R and E residues (e.g., R4–E21) are presented as key drivers of AH–AH association and anti-parallel orientation. However, the analysis remains largely qualitative:
• Please provide quantitative occupancies (e.g., fraction of simulation time where each salt-bridge pair is formed) and associated lifetimes across all interacting replicas.
• Consider including radial distribution functions or contact probability matrices summarizing all charged residue pairs.
• It would be helpful to discuss how sensitive these salt-bridge patterns are to the chosen distance cutoffs and whether alternative definitions change the conclusions.
Given how central these interactions are for the proposed cooperative mechanism, a more rigorous statistical treatment is required.

<u>Authors' Reply:</u> We agree that our original discussion of salt bridges was largely qualitative. To provide a more rigorous and quantitative characterization, we have added three complementary analyses: (i) representative time traces of charged residue–pair distances from the VMD salt-bridge tool for interacting replicas (now Fig. S10A), (ii) a systematic trajectory-based occupancy analysis across all eight replicas (Table S1 and Fig. S10B), and (iii) salt-bridge lifetime statistics (continuous dwell-time events) for the same set of residue pairs (Table S2). In the MDAnalysis-based analysis, a salt bridge is defined using a standard charged-group distance criterion with a 3.5 Å cutoff.

The occupancy analysis (Table S1; Fig. S10B) shows that salt bridges are intermittent and heterogeneous across replicas (reflected by the larger standard deviations), yet a small subset of residue pairs accounts for the majority of observed inter-peptide electrostatic contacts. Notably, although the peptide contains only two arginine residues (R2 and R4) (compared to seven lysines), Arg-involving pairs repeatedly appear among the most populated contacts (e.g., R4–E10, R4–E21, R4–E20, R2–E10). This indicates that Arg-mediated salt bridges contribute a substantial share of the detected salt-bridge interactions relative to the limited number of Arg sites, consistent with an enriched role for these residues during AH–AH association.

The lifetime analysis (Table S2) further demonstrates that these interactions are not limited to single-frame encounters but recur as multi-frame events. For example, high-contributing pairs such as K18–E10 and R4–E10 show many events (40–50) and the largest total "on-time" (321 ns and 280 ns, respectively), while R4–E20, although less frequent, exhibits longer-lived episodes (median 18 ns; 95th percentile 73.8 ns). Together, the added occupancy and lifetime statistics provide a quantitative foundation for the charged interactions discussed in the manuscript.

Please see pp. 15, 16, 47, 48

6.The Data Availability statement indicates that full trajectories will be provided "upon reasonable request" due to storage and bandwidth limitations, whereas only processed data and representative frames are provided in the Supplementary Materials.
• Please clarify whether full or at least subsampled trajectories can be deposited in a public repository (e.g., Zenodo, OSF, or a domain-specific MD repository) with appropriate compression or frame thinning.
• If only a subset can be shared, specify clearly in the Data Availability statement what exactly is available, where, and how it suffices to reproduce all figures and key analyses.

<u>Authors' Reply:</u> We thank the reviewer for this suggestion. We agree that open access to the simulation data is valuable for transparency and reproducibility. We deposit the full simulation trajectories and associated input files in an open-access repository (Zenodo), as indicated in the Data Availability section (p.33).

7.The manuscript repeatedly references prior work from the authors on isolated AH domains and curvature sensing. It is sometimes difficult to distinguish which insights are genuinely new versus extensions or re-analyses of previously published simulations.

• Please more explicitly contrast the present work with previous studies, clearly stating what data and analyses are novel here (e.g., extended AH with flanking regions, explicit tandem constructs, two-peptide cooperative effects).
• Consider adding a short paragraph in the Introduction or Discussion that lists the main conceptual and technical advances beyond the prior study.
This clarification is important for assessing the incremental contribution of the current manuscript.

Authors' Reply: We thank the reviewer for highlighting the need to more clearly delineate the novelty of the present study. In response, we have added a new paragraph to the Introduction explicitly contrasting this work with our previous studies of isolated AH domains. We now clearly state the key conceptual and technical advances introduced here, including the use of extended AH constructs with conserved flanking regions, explicit tandem AH simulations, and multi-peptide systems that reveal cooperative peptide–peptide and peptide–lipid effects not accessible in prior single-helix analyses, to emphasize on novel contributions of the current manuscript. We have also reviewed the manuscript to ensure that references to prior work are clearly framed as context, while new results and analyses are explicitly identified as novel (p. 4).

8.The APL and lipid order-parameter maps for single vs. double peptide systems are interesting, but the interpretation is again largely qualitative.
• Please provide quantitative statistics (mean ± SD over time and replicas) for APL and order parameters in defined regions (e.g., within a given radius of peptide vs. distal bulk membrane).
• Some of the proposed functional interpretations (e.g., "lateral pressure" leading to peptide displacement, or specific defect patterns being more compatible with curved membranes) would benefit from more cautious wording unless supported by additional analysis (e.g., lateral pressure profiles, free-energy calculations, or comparisons with curved systems).

Authors' Reply: We thank the reviewers for their suggestion. We quantified area per lipid (APL) and the lipid nematic order parameter (S) in two lateral regions: Near (≤ 2.0 nm from the peptide) and Bulk (≥ 4.0 nm, distal membrane). Results are reported as mean ± SD over replicas, where each replica contributes its time-averaged value (over the analyzed trajectory window).

Area-per-lipid
Near: 0.6955 ± 0.0052 nm^2
Bulk: 0.6823 ± 0.0031 nm^2
Near vs Bulk: paired t-test $p = 0.0436$

Nematic order parameter, S:
Single peptide — Near: 0.111656 ± 0.000922
Single peptide — Bulk: 0.130725 ± 0.002988
(Single peptide, Near vs Bulk: paired t-test $p = 0.000636$)

Double peptide — Near: 0.092094 ± 0.003865
Double peptide — Bulk: 0.130717 ± 0.002516
(Double peptide, Near vs Bulk: paired t-test $p = 0.000912$)

All the results were assessed to be statistically significant ($p<0.05$).

We agree that mechanistic interpretations should be stated more cautiously. We revised the text throughout the Abstract, Author Summary, Results, and Discussion to describe the observed displacement and membrane remodeling as correlations with altered packing (supported by the new Near vs Bulk APL and nematic-order statistics), and we removed or softened statements attributing these effects to lateral pressure or implying preferential compatibility with curved membranes. We now explicitly note that curvature-related implications are hypotheses that will require more directed experiments to confirm.
Please see p. 25

Minor Comments

1.The Abstract and Author Summary are generally clear but somewhat dense, with several long sentences that mix methodological details and mechanistic conclusions. Consider tightening the text to distinguish more clearly between observed simulation results and proposed broader implications.

Authors' Reply: We thank the reviewer for this helpful suggestion. We have revised the Abstract and Author Summary to improve clarity and readability (pp. 1-2).

2.The manuscript uses several terms such as "extended AH," "floating peptide," and "bound peptide" that are not consistently defined at first use. A concise table or schematic summarizing all constructs (single AH, extended AH, NC–NC tandem, CN–NC tandem, extended CN–NC, etc.) and their sequences would improve readability.

Authors' Reply: We thank the reviewer for this suggestion. We have revised the Introduction to explicitly define the terms "floating peptide" and "bound peptide" at first use and to link these definitions directly to Fig. 1. Throughout the manuscript, these terms are now used consistently. The various peptide constructs (extended AH and tandem constructs) are also described explicitly in the Methods section (p. 5).

3.There are several minor typographical errors (e.g., occasional misspellings and missing articles). A careful proofreading or copy-editing pass is recommended to improve polish.

Authors' Reply: We thank the reviewer for noting this. We have carefully proofread the manuscript and corrected typographical errors, misspellings, and minor grammatical issues throughout the text as part of the revision.

4.Some figure panels (e.g., curvature heatmaps and multi-panel supplementary figures) have small fonts and color scales that are difficult to read, especially in print. Please ensure that axis labels, legends, and color bars are clearly legible at journal print size and that color choices are accessible (e.g., for color-blind readers).

<u>Authors' Reply:</u> Thank the reviewer for this suggestion. We have revised the main-text figures, including the curvature heatmaps, Fig.2, to improve font sizes. All figures are provided at full resolution in the online version.

5.The sequence logo and alignment used to argue for conservation of E and R residues involved in salt bridges are useful, but the Methods should provide more detail (number of sequences, selection criteria, species coverage) and the figure legend should explicitly mark the key residues discussed in the text.

<u>Authors' Reply:</u> We thank the reviewer for this helpful suggestion. We have revised the Methods to include additional details on the number of sequences analyzed, selection criteria, and alignment procedure used for the sequence analysis. We have also revised the Figure 6 caption to explicitly identify the conserved glutamic acid and arginine residues involved in the salt-bridge interactions described in the simulations and have clearly marked these residues in the alignment (p.22, 31).

6.The Methods specify specific ionic strength and lipid composition. It would be helpful to briefly justify these choices in the context of known yeast membrane composition and ionic strength, and to comment on how variations in composition or salt might alter the observed behavior.

<u>Authors' Reply:</u> We thank the reviewer for this suggestion. We have revised the Methods to briefly justify the chosen lipid composition and ionic strength as a minimal, biologically motivated model that captures key features of the yeast plasma membrane, including enrichment in anionic lipids relevant for septin binding. We also added a short discussion noting that variations in lipid composition or salt concentration are expected to modulate the balance of electrostatic and hydrophobic interactions, but are not expected to qualitatively alter the cooperative peptide–peptide and peptide–lipid interaction mechanisms identified here (pp. 11, 24, 28).

7.The Bendix analysis uses a specific segment length to define local curvature. The text mentions a trade-off between resolution and smoothness, but it would be useful to show that the qualitative bending pattern is insensitive to moderate variations in this parameter (e.g., in supplementary information).

<u>Authors' Reply:</u> We thank the reviewer for this helpful suggestion. In our analysis, the Bendix segment length was chosen to balance spatial resolution and smoothness and is appropriate for the length of the extended AH domain studied. Our conclusions are based on qualitative features of the bending profile, such as the asymmetric curvature and the location of maximal bending, rather than on absolute curvature values. As such, moderate variations in segment length are not expected to qualitatively alter the observed bending pattern. We have clarified in the Methods that our conclusions rely on qualitative bending patterns rather than absolute curvature values (p. 31).
